# Milankovitch-paced erosion in the southern Central Andes

**DOI:** 10.1038/s41467-023-36022-0

**Published:** 2023-01-26

**Authors:** G. Burch Fisher, Lisa V. Luna, William H. Amidon, Douglas W. Burbank, Bas de Boer, Lennert B. Stap, Bodo Bookhagen, Vincent Godard, Michael E. Oskin, Ricardo N. Alonso, Erik Tuenter, Lucas J. Lourens

**Affiliations:** 1 Jackson School of Geosciences, University of Texas at Austin, Austin, TX 78712 USA; 2 Earth Research Institute, University of California, Santa Barbara, CA 93106 USA; 3 Institute of Environmental Science and Geography, University of Potsdam, Potsdam, Germany; 4 Department of Earth and Climate Sciences, Middlebury College, Middlebury, VT 05753 USA; 5 Earth and Climate Cluster, Faculty of Science, Vrije Universiteit Amsterdam, Amsterdam, the Netherlands; 6 Institute for Marine and Atmospheric Research, Utrecht University, Utrecht, the Netherlands; 7 Institute of Geoscience, University of Potsdam, Potsdam, Germany; 8 Aix-Marseille Univ., CNRS, IRD, INRAE, CEREGE, Aix-en-Provence, France; 9 Institut Universitaire de France, Paris, France; 10 Department of Earth and Planetary Sciences, University of California, Davis, CA USA; 11 Universidad Nacional de Salta, Salta, Argentina; 12 Royal Netherlands Meteorological Institute (KNMI), De Bilt, the Netherlands; 13 Department of Earth Sciences, Faculty of Geosciences, Utrecht University, Utrecht, the Netherlands

**Keywords:** Palaeoclimate, Hydrology, Geomorphology, Sedimentology, Tectonics

## Abstract

It has long been hypothesized that climate can modify both the pattern and magnitude of erosion in mountainous landscapes, thereby controlling morphology, rates of deformation, and potentially modulating global carbon and nutrient cycles through weathering feedbacks. Although conceptually appealing, geologic evidence for a direct climatic control on erosion has remained ambiguous owing to a lack of high-resolution, long-term terrestrial records and suitable field sites. Here we provide direct terrestrial field evidence for long-term synchrony between erosion rates and Milankovitch-driven, 400-kyr eccentricity cycles using a Plio-Pleistocene cosmogenic radionuclide paleo-erosion rate record from the southern Central Andes. The observed climate-erosion coupling across multiple orbital cycles, when combined with results from the intermediate complexity climate model CLIMBER-2, are consistent with the hypothesis that relatively modest fluctuations in precipitation can cause synchronous and nonlinear responses in erosion rates as landscapes adjust to ever-evolving hydrologic boundary conditions imposed by oscillating climate regimes.

## Abstract (web summary)

Fisher et al. combine sediment geochemistry and climate modelling to reveal long-term synchrony between erosion rates and orbitally-driven climate oscillations in the tectonically-active southern Central Andes.

## Introduction

The hypothesis that climate exerts a first-order control on landscape form and function through physical and chemical erosion dates back more than a century^1^. Nonetheless, quantifying the influence of climate on erosion rate and morphology in any given landscape has proven difficult. Whereas numerical models provide clear rationales for how precipitation foci and oscillations may impact deformation and sediment-flux regimes in orogenic systems^2–5^, empirical evidence has remained equivocal with convincing evidence both for and against strong coupling between climate, erosion, and tectonics^6^. This ambiguity partially derives from temporal and spatial mismatches in both the datasets and proxies used to infer such linkages. For example, many interpretations rely on comparing present-day precipitation patterns with erosion and/or tectonic rates measured over 10^2^- to 10^6^-year timescales. Further complications arise in deciphering the primary driver of erosion in orogenic systems due to the common spatial coincidence between precipitation, topographic relief maxima, and deformational foci as a consequence of orographic effects^7^.

Acknowledging these complications, researchers have sought to exploit sedimentary archives in order to extract temporal records of erosion rates for direct comparison to known oscillations and excursions in climate^8–25^. Unfortunately, these studies are rarely straightforward, and commonly rely on less-than-ideal field exposures, temporal controls, and source-area and cosmogenic radionuclide systematic constraints. In addition, many studies lack the sampling frequencies necessary to unambiguously link paleo-erosion-rate trends with climatic changes. For example, in the Tien Shan region, the addition of new sediment archives resulted in the revised conclusion that tectonics was predominantly responsible for controlling erosion in the region during the Plio-Pleistocene^16^ rather than the onset of Quaternary glaciation as previously proposed^11^. Likewise, in the south-central Andes of Argentina, two studies developed paleo-erosion rate records along the same section^14,18^. Denser sampling and improved temporal controls used by the later study^18^ allowed identification of two pronounced reductions in paleo-erosion rates not identified by the earlier study and consistent with regional and marine climate proxies.

Despite the complexities involved with extracting high-quality erosion-rate data from terrestrial sediment records, such data have the potential to provide unique insights into how landscapes respond to diverse climatic and tectonic perturbations across a range of forcing frequencies. To date, considerable research effort has focused on identifying Milankovitch cycles, commonly referred to as precession (~23 kyr cyclicity), obliquity (~41 kyr), and eccentricity (~100 kyr and ~400 kyr), within terrestrial sedimentary records. These orbital periodicities, which change the latitudinal distribution of solar insolation by season, have been widely acknowledged across marine and atmospheric records as key global climate drivers. Yet, linkage of sediment fluxes or erosion rates to these climate drivers over multiple cycles has remained largely theoretical, especially in active tectonic settings^2,4,5^. Thus, any direct linkage between climate oscillations and sediment flux from the continents could have far-reaching implications for landscape evolution theory, chemical weathering regimes, and ecosystem and evolutionary trajectories^26,27^.

**Fig. 1:**
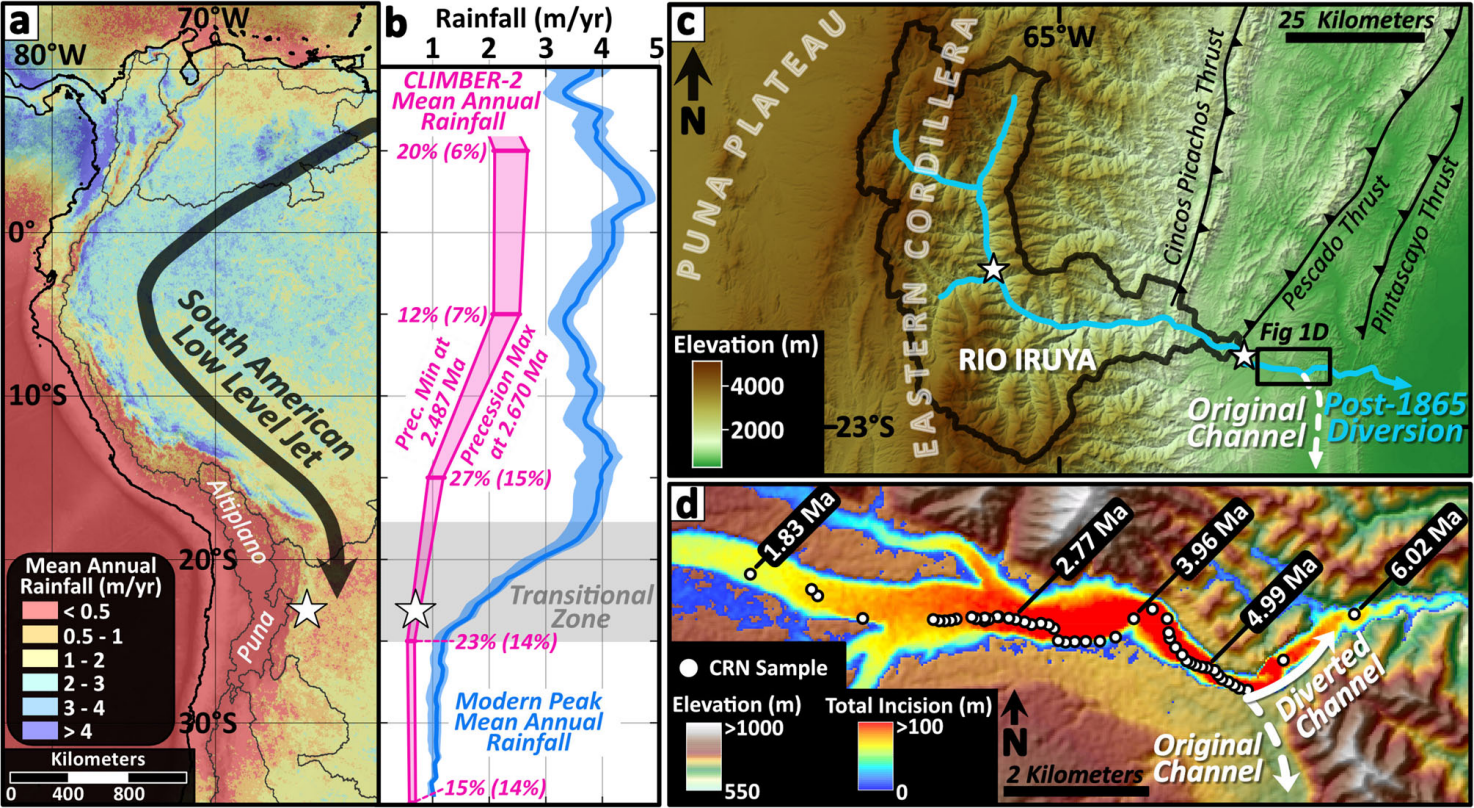
Geographic and climatic setting of the Río Iruya study area. **a** Major modern South American climate and topographic components including modern mean annual rainfall derived from the Tropical Rainfall Monitoring Mission (TRMM) 2B31 dataset^7^, the South American low-level jet (SALLJ), and the internally-drained, high-elevation Altiplano and Puna plateaus (see Supplementary Fig. 1 for detailed climatic and topographic characteristics of the study area). The white star locates the study site in **a** and **b**. **b** Plot of modern peak mean annual rainfall (±2*σ* envelope) (blue) extracted from 115, 50-km-wide by 1000-km-long, orogen-perpendicular swath profiles along the Andes (see^7^) indicating a transitional zone (gray) between ~18– 25°S latitude. Linearly-interpolated mean annual rainfall derived from CLIMBER-2 model results (magenta), extracted from a longitudinal swath spanning 95 − 115°W at 10° latitude intervals from 10°N to 35°S in the model (see Methods for discussion of the geographic offset of the model), shows maximum precipitation values from a precession maximum at 2.670 Ma and minimum values from a precession minima at 2.487 Ma. Magenta percentages indicate the maximum percent increase in mean annual precipitation between the plotted precession minimum and maximum values, with the mean differences in parentheses. **c** Overview of the Río Iruya watershed geography and Eastern Cordillera topography with predominant thrust faults labeled. Locations of modern cosmogenic radionuclide (CRN) erosion-rate samples (IR-3M and IR-6M) are shown as white stars. **d** Close-up of the Río Iruya canyon section showing the pre- and post-diversion Río Iruya flow directions (hashed and solid white arrows, respectively), CRN paleo-erosion rate sample locations (white circles) with select stratigraphic ages (white numbers), and total estimated incision in the canyon over the ~150 years post diversion (blue-red color gradient).

To address the hypothesis that long-term variations in climate can drive consequent responses in erosion rate, we present 49 ^10^Be and 23 ^26^Al cosmogenic radionuclide-derived paleo-erosion rates sampled along the Río Iruya canyon in northwest Argentina spanning the period from 6-1.8 Ma (Fig. 1 and Supplementary Figs. 1 to 3). This approach to calculating paleo-erosion rates is similar to the well-established practice of calculating erosion rates (10^2^−10^4^-year timescales) from present-day cosmogenic radionuclide concentrations in river sediments, which quantifies the combined signal (also referred to as denudation) from both physical erosion and chemical weathering within the contributing catchment^28^. The technique relies on the fact that cosmogenically produced nucleons attenuate exponentially with depth through the upper few meters of rock and regolith, where they spall O and Si atoms in quartz into ^10^Be (half-life = 1.387 Myr)^29^ and ^26^Al (half-life = 0.705 Myr)^30^, respectively, which accumulate over time. This behavior permits radionuclide concentrations in surface samples to be converted into erosion rates, whereby the concentration is inversely proportional to the rate^31^. Assuming that the fluvial system reliably integrates sediment from throughout the catchment, a sample of river sediment provides an accurate estimate of the mean catchment erosion rate^28^. If these sediments are then rapidly buried and progressively preserved over millions of years, a time-resolved record of paleo-erosion rates can be reconstructed by measuring ^10^Be or ^26^Al in quartz from sediment in a well-dated depositional sequence^10,11,14,17,18^.

**Fig. 2:**
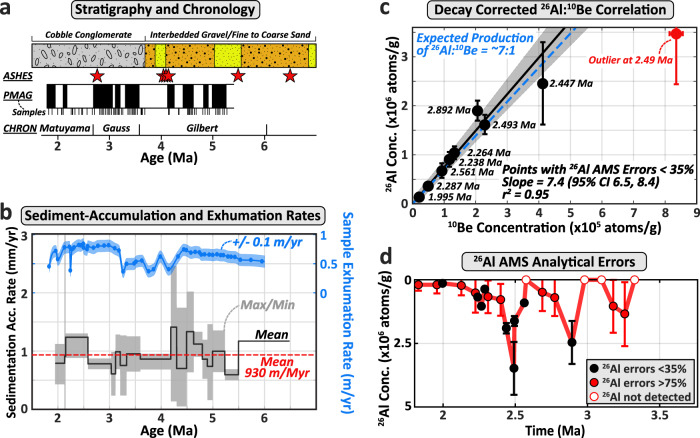
Advantageous characteristics of the Río Iruya canyon section for cosmogenic radionuclide paleo-erosion rate analysis. **a** Stratigraphy, volcanic ashes with high-resolution ages (red stars), magnetostratigraphic (PMAG) sample sites, magnetic polarity (black = normal polarity; white = reversed polarity), and overall chronologic constraints for the Río Iruya section^32,34^ (see Supplementary Fig. 2 and Supplementary Dataset 1). **b** Calculated sediment-accumulation rates for the Río Iruya section^32^ (mean in black and maximum and minimum envelope in gray). Dashed red line is the overall mean accumulation rate for the section. Rate uncertainties are greatest for brief magnetochrons where the precise positions of polarity boundaries are not well resolved relative to the length of the magnetochron (see Supplementary Dataset 1). Mean exhumation rates for cosmogenic radionuclide samples (blue dots and trend line) are based on the sample depth below the pre-diversion river level and assuming 150 years since the diversion. The associated error envelope assumes a+/− 15 m error on the reconstructed pre-diversion surface elevation. **c** An error-weighted two-nuclide test for complex transport and burial history showing minimal diversion from the expected decay-corrected ^26^Al:^10^Be ratio (7:1 in dashed blue line) for all ^26^Al samples with detectable concentrations and AMS errors less than 35%, minus the outlier at 2.49 Ma (95% confidence intervals are shown by the gray envelope; *n* = 8; mean ratio = 7.4:1). Large ^26^Al errors are related to dilution of the ^26^Al/^27^Al ratio by considerable inherent Al in the sample quartz. Note ^26^Al and ^10^Be errors are based on the paleo-erosion concentration component, N_E_ (i.e., corrected for exhumation, decay, and burial components), with 2*σ* errors reported (^10^Be errors fall within the markers). **d** Plot of ^26^Al errors with sediment age and concentration showing the non-systematic distribution of high AMS error samples (>75%).

The Río Iruya canyon, located on the eastern flank of the northern Argentinian Andes and with a watershed bordering the internally-drained central Andean Plateau (Puna Plateau), provides an ideal location to apply a cosmogenic radionuclide paleo-erosion rate technique for three reasons (Fig. 1). First, the close proximity of the depositional foreland basin to a steep source area (<100 km) minimizes the likelihood of prolonged sediment storage, which may alter ^10^Be concentrations acquired in the target catchment during source-to-sink transport. Second, rapid foreland-basin deposition rates (~1 m/kyr)^32^ suggest that ^10^Be concentrations at deposition have been minimally affected by post-depositional nuclide accumulation during burial (Fig. 2). Lastly, the Río Iruya canyon is the result of a large-scale flood-diversion project completed in 1865 that led to the rerouting and shortening of the mainstem Río Iruya (Fig. 1c). Subsequent vertical incision of ~100 m has recently exposed >8 km of continuous Miocene-to-Pleistocene-aged foreland sediments known as the Oran Group^32–34^. The rapid exhumation of the canyon (0.5–1 m/yr) minimizes ^10^Be production due to recent exposure and, when combined with a continuous, high-resolution magnetochronology^32^ and a simple pre-burial transport history (Fig. 2 and Supplementary Fig. 2), permits extraction of a robust record of erosion-rate variability that minimizes confounding and commonly unconstrained sources of post-depositional ^10^Be and ^26^Al.

**Fig. 3:**
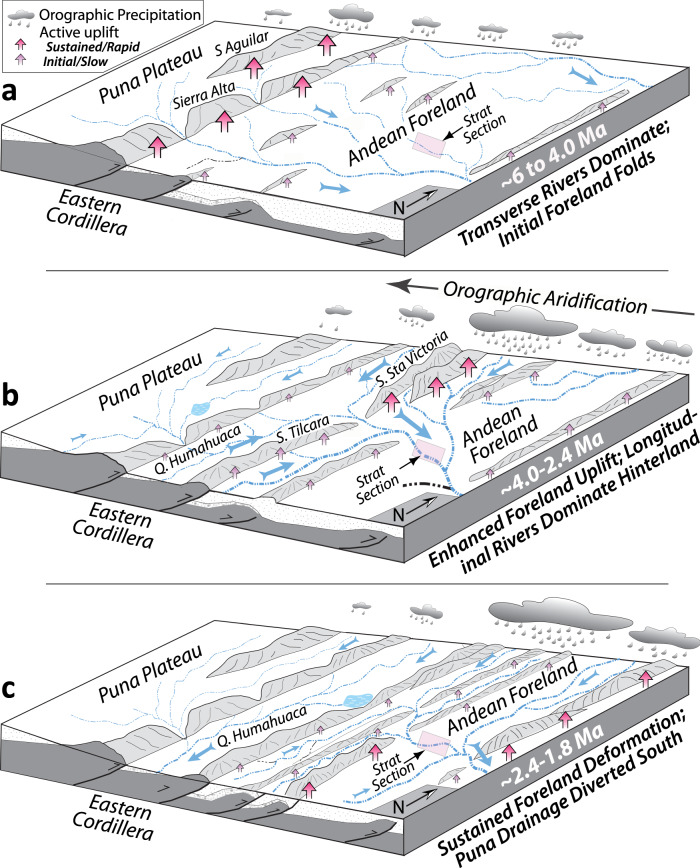
Schematic illustration of late Miocene-to-Pleistocene tectonics, climate, and deposition in the Río Iruya catchment. Pink rectangle indicates the approximate location of the Río Iruya section of this study. **a** Latest Miocene to mid-Pliocene interval featuring ongoing uplift of the Eastern Cordillera and early stages of foreland development. Largely transverse drainages dominate the depositional system and Río Iruya watershed may not have been directly connected to the eastern Puna Plateau. **b** Mid-Pliocene-to-Early Pleistocene period characterized by ongoing tectonic uplift in the eastern Cordillera (Sierra Santa Victoria and Sierra Tilcara). The Río Iruya watershed may have been connected to the eastern Puna Plateau based on the strong proportion of Neogene-aged zircons in the sediment. **c** Hinterland Puna Plateau drainages such as the Quebrada de Humahuaca are fully diverted to the south by sustained range growth and increasing aridification. The Río Iruya may have become disconnected from the Puna Plateau and begun to establish its modern extent based on the near disappearance of Neogene zircons from the sediment.

The Río Iruya watershed drains the eastern edge of the Puna Plateau, which is defined by a series of mountain ranges known as the Eastern Cordillera. In the Iruya watershed, the Eastern Cordillera exhibits nearly 4000 m of relief, plunging steeply downward into the adjacent foreland basin, which is defined by a N-S striking fold and thrust belt (Fig. 1). The Eastern Cordillera is likely growing over a footwall ramp, which extends eastward as a detachment surface with splay faults connecting upwards to several foreland faults and folds^33,35^. In the study region, uplift of the eastern Puna Plateau was likely underway by the Oligocene^36,37^, creating significant relief, deep basins and orographic barriers by ca. 10–8 Ma^35,38,39^. Facies migration within regional foreland basin sequences suggests the onset of thrusting in the Eastern Cordillera at roughly 15 Ma^40^ consistent with the onset of deposition of the Oran Group between ca. 13.5–8 Ma^33,34,41,42^. Uplift and erosion of the Eastern Cordillera has continued until today, resulting in progressive incision of the Río Iruya into the metamorphic core of the Eastern Cordillera and with its headwaters tapping into basins of the eastern Puna Plateau (Fig. 3)^43^. A detrital fission-track study along the Río Iruya suggests that areas of peak incision (e.g., the Río Astillero) have experienced roughly 6 km of exhumation over the last 10 Myr, yielding a long-term average rate of roughly 0.6 mm/yr^43^.

Amidon et al.^32^ have argued that the Río Iruya watershed experienced major provenance shifts at roughly 4.0 Ma and 2.4 Ma, each of which corresponds temporally with changes in the spatial pattern of thrust faulting and surface uplift^32^. The first provenance shift is recorded by the addition of Neogene zircons as well as a very restricted range of single-grain quartz chemistries occurring between 4.3 and 3.5 Ma^32^. The observed increase of Neogene-aged zircons is interpreted to result from the watershed gaining connection to a source of sediments from the volcanic provinces of the eastern Puna Plateau. The restricted quartz chemistry is interpreted as a more localized sediment source. Together, these observations suggest a change in the extent of the Río Iruya watershed during this period. This shift is roughly contemporaneous with out-of-sequence deformation in the foreland between 4.5 and 4.0 Ma as thrusting stepped backwards onto the Pescado Thrust (Fig. 1)^33,34^. Meanwhile, in the Humahuaca region just to the southwest, active thrusting along the eastern edge of the Puna Plateau disconnected the basin from the foreland, rerouting fluvial networks into a more N-S axial geometry and triggering deposition of the fluvial Tilcara Formation by roughly 4.2 Ma (Fig. 3)^44^. Likewise, the nearby Sierra Alta experienced a pulse of uplift at around 4.3 Ma (Fig. 3)^45^.

The second provenance shift occurred between 2.4 and 2.1 Ma as the proportion of Neogene zircons drops by ~90% and the distribution of single-grain quartz chemistry regains its full complexity^32^. This provenance change corresponds to an important stratigraphic boundary as the Terciario Subandino sequence is unconformably overlain by the coarse El Simbolar conglomerates^33,34^. This provenance shift is likely related to ongoing tectonic deformation in the region, which eventually caused another change in the watershed geometry. For example, foreland deformation stepped out to the easternmost Aguaragüe fault at roughly 2.7 Ma^33^. In the Humahuaca region (Fig. 3), increasing aridification caused denudation rates to drop by an order of magnitude from ~4 Ma to 2.7 Ma^21^, and deposition of the fluvial Tilcara formation ceased, giving way to coarse alluvial conglomerates by 2.4 Ma^44^.

Climatically, the modern Río Iruya region has a semi-arid subtropical climate in which precipitation is heavily controlled by topography, with mean annual precipitation varying from <0.5 m/yr on the arid Puna Plateau to ~2.5 m/yr on high-relief, east-facing mountain slopes^7^ (Fig. 1, Supplementary Fig. 1). Nearly all precipitation is derived from easterly or northeasterly sources due to the topographic barrier of the Puna Plateau and hyper-arid Atacama Desert immediately to the west. Roughly 80% of precipitation in the study region falls in the Austral summer months between November and February^46^ when easterly winds bring moisture off the Atlantic Ocean, fueling the South American Monsoon System (SAMS) over Brazil^47^. Some of this moisture is channeled down the Andean Front to the Río Iruya watershed via the South American Low Level jet (SALLJ): a low-level atmospheric flow drawn toward the Chaco high pressure system near 25° S (Fig. 1a). Given its location in the continental interior and the highly seasonal precipitation, precipitation in the Río Iruya region is sensitive to the strength and position of the South American monsoon and the SALLJ, which are in turn governed by patterns of global oceanic and atmospheric circulation^48^.

Although several studies suggest the SALLJ system was in place by the mid-Miocene^39,49^, very little is known about the terrestrial climatic conditions in the Andes during the Pliocene-Pleistocene time given the lack of high-resolution lacustrine or glacial records during this period. We thus assess the role of climatic forcing using the low-resolution, intermediate complexity CLIMBER-2 model^50^. The CLIMBER-2 model, although coarse in spatial resolution (latitudinal resolution of 10° and longitudinal resolution of ~51°) and insensitive to regional orographic forcings and climate systems driven by the topography of the Andes (e.g., the SALLJ), provides one of the only robust modeling tools with which to perform long-term climate system simulations over millions of years. To this end, the model provides a broad understanding of the magnitude and frequency of hemispheric moisture transport to the study region during the Plio-Pleistocene.

In this work, we document the direct erosional response of a tectonically-active southern Central Andes watershed (Río Iruya) to imposed climate oscillations during the Pliocene, as well as to a major climate transition at the Plio-Pleistocene boundary. We propose that the 400-kyr eccentricity-paced erosion-rate signal observed during the Pliocene arises from nonlinear fluvial incision and hillslope erosion thresholds, namely those associated with landsliding in the catchment: a signal resolvable in the sedimentary record because of the erosional sensitivity of the study region to relatively small perturbations in precipitation. We posit that the observed synchrony between erosion rates and climate oscillations in this unique study area is likely restricted to those regions dominated by transitional climate regimes (i.e., insolation-driven monsoons) and where geomorphic response times and processes are sensitive enough to record the imposed climatic oscillations (e.g., arid to semi-arid climates). These findings should newly inform ongoing debates concerning the role of climate cyclicity in driving landscape evolution, as well as the extent to which nonlinearities in the geomorphic response of a landscape may destroy, dampen, or enhance climate signals across diverse climate-erosion-tectonic couplings and those recorded in sedimentary archives.

## Results

**Fig. 4:**
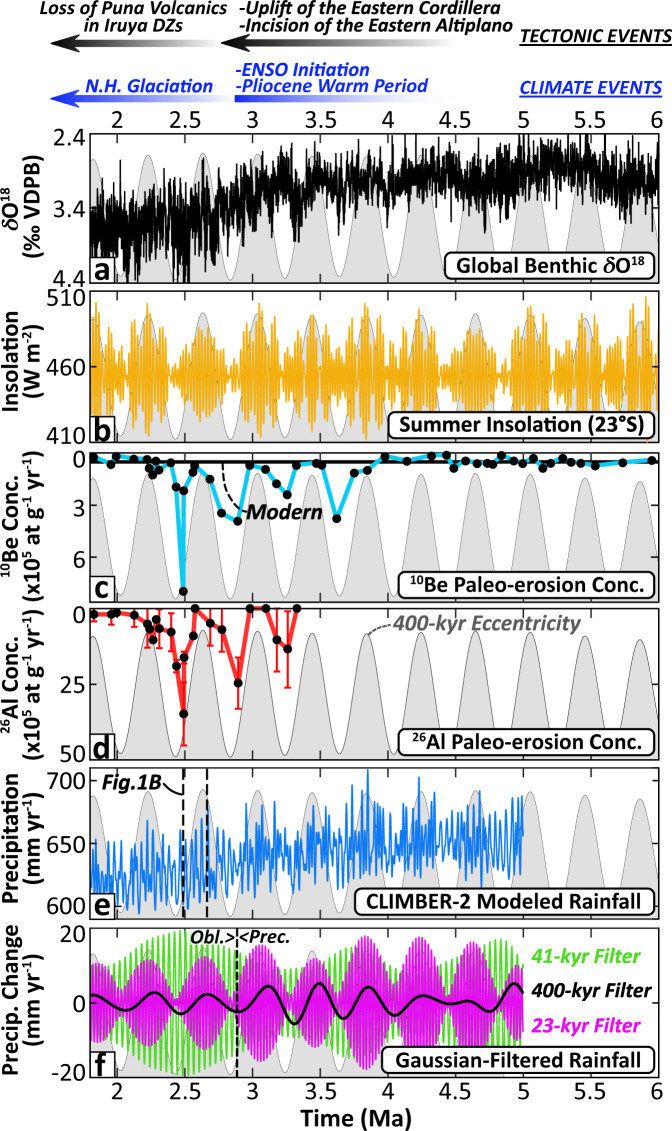
Cosmogenic radionuclide concentrations and climate datasets. **a** Global compilation of benthic δ^18^O values^51^ (VDPB, the Vienna Pee-Dee belemnite standard) and **b** mean austral summer insolation (Dec.-Mar.) calculated at the 23°S latitude of the Río Iruya study area^83^. **c** ^10^Be and **d** ^26^Al paleo-erosion concentrations (N_E_ - i.e., corrected for exhumation, decay, and burial components) (2*σ* errors are contained within the black circles for ^10^Be) on reversed axes where decreasing concentration is indicative of increasing erosion rate, assuming a constant mean catchment-production rate. **e** Modeled mean annual precipitation rate for the Río Iruya region (taking the grid box that covers the region of interest, i.e., from 116.4–65°W and 30-20°S) derived from the CLIMBER-2 model with **f** the Gaussian-filtered 23-kyr (magenta), 41-kyr (green), and 400-kyr (black) CLIMBER-2 precipitation components. Note: the 400-kyr eccentricity curve is shown in light gray and repeated across all panels for reference. N.H. = Northern Hemisphere; DZs = detrital zircons; ENSO = El Niño Southern Oscillation; Obl. = obliquity; Prec. = precession.

**Fig. 5:**
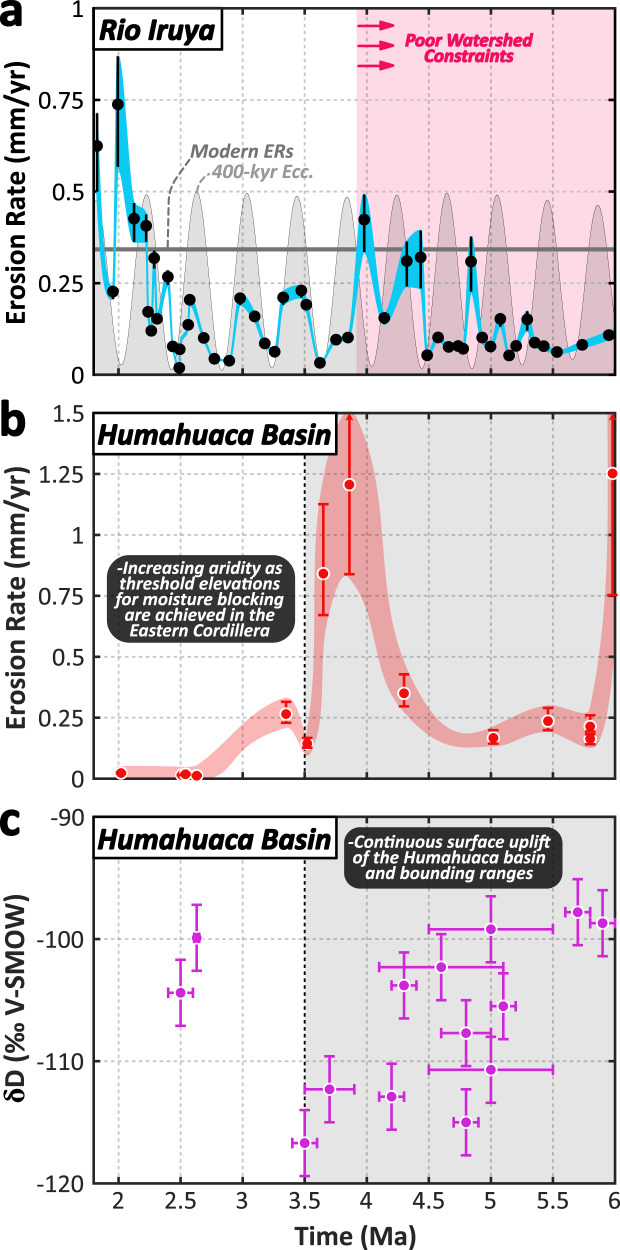
^10^Be-derived paleo-erosion rates from the Río Iruya and nearby Quebrada de Humahuaca from 6 Ma to 1.8 Ma. **a** Paleo-erosion rates calculated for the Río Iruya catchment based on the preferred uplift regime and mean catchment production rate evolution scenario (see Supplementary Fig. 4b and Supplementary Dataset 2). Black circles indicate samples, and the blue piecewise cubic spline is interpolated between sample errors (±2*σ* envelope). Long-eccentricity cycles (400 kyr) are shown in the background for reference (light gray) with the modern Río Iruya catchment erosion rate (0.34 mm/yr) shown in dark gray. **b** Paleo-erosion rates (±1*σ* bars)(red) and **c** δD values of volcanic glass shards sampled throughout the adjacent intermontane Quebrada de Humahuaca (purple) (±1*σ* on age and δD values) (Fig. 3, Supplementary Fig. 3)^21,86^. Data from the Humahuaca Basin document sustained surface uplift of the Humahuaca Basin and surrounding ranges in the Eastern Cordillera from ~6 Ma to 3.5 Ma resulting in the development of a significant orographic barrier along the Eastern Cordillera by 3.5 Ma. Continued orographic isolation and aridification of the basin followed, driving an order of magnitude reduction in basin denudation rates despite the presence of considerable topography. V-SMOW - Vienna standard mean ocean water.

Paleo-erosion rates spanning approximately 6–1.8 Ma can be broadly grouped into three periods of behavior spanning 6–4 Ma, 4–2.4 Ma, and 2.4–1.8 Ma (Fig. 4 and Fig. 5). Although these intervals are defined by the nature of the erosion-rate record, transitions between them are roughly contemporaneous with previously documented tectonic and sediment provenance shifts at ~4.0 and ~2.4 Ma described above. All paleo-erosion concentrations (N_E_) and paleo-erosion rates (R_E_) presented have been corrected for exhumation (N_X_), decay (N_D_), and burial components (N_B_) (see Methods for details). Outside of the decay component, the burial component provided the largest modification of the paleo-erosion concentrations used to infer erosion rates (N_E_) with values ranging from 1–36% (mean = 10%). Concentration modification associated with exhumation was negligible (<0.05%) as expected given the recent, rapid incision of the Río Iruya canyon.

### Paleo-erosion Rates from 6 Ma to 4 Ma

Prior to ~4 Ma, erosion rates vary widely from ~0.05 to 0.42 mm/yr based on the assumptions of the preferred catchment-uplift and production-rate evolution scenario (Fig. 5, Supplementary Fig. 4). Despite the large number of paleo-erosion rate estimates (*n* = 21), it is difficult to attribute a consistent pattern or frequency to the erosion rates during this time interval, especially given that many of the erosion-rate peaks are defined by single data points. It seems unlikely that this ambiguous erosion-rate pattern is due to uncertainty in stratigraphic age given that the assigned stratigraphic ages are tightly constrained by 8 magnetic reversals spread throughout the period from 4.2 to 5.3 Ma and anchored by four volcanic ash beds (Fig. 2, Supplementary Fig. 2). Although the geometry of the watershed prior to 4 Ma is uncertain, field observations and provenance studies indicate deposition during this period occurred in a larger and more distal depositional system characterized by finer-grained sediments and a poorly-constrained source area (Fig. 3a)^32^.

### Paleo-erosion Rates from 4 Ma to 2.4 Ma

**Fig. 6:**
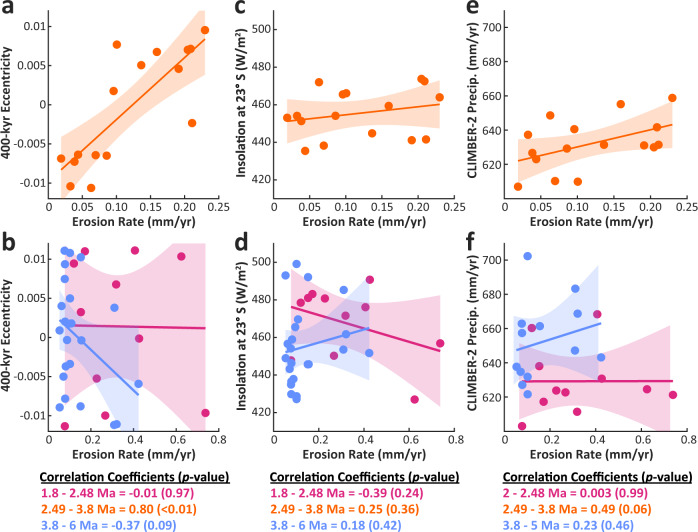
Pearson’s correlation between erosion rates and climatic parameters. Erosion rates compared to **a**, **b** 400-kyr eccentricity, **c**, **d** insolation at 23° S latitude, and **e**, **f** the CLIMBER-2 precipitation dataset for three different time periods: 1.8–2.48 Ma (red), 2.49–3.8 Ma (orange), and 3.8–6 Ma (or 5 Ma for the CLIMBER-2 dataset) (blue). Pearson’s correlation coefficients and *p*-values are presented for three periods of time below each plot with 95% confidence interval envelopes shown for each correlation. Note the strong correlation and low *p*-values for the relationships between erosion rate and 400-kyr eccentricity and CLIMBER-2 precipitation for the period from 2.49–3.8 Ma (orange) in **a** and **e**. Also note the different x-axis scalings between the upper and lower panels.

At ~4 Ma, ^10^Be concentrations record the onset of a pronounced periodicity in erosion rates from ~4 to 2.4 Ma (Fig. 4 and Fig. 5). This period is characterized by up to three complete paleo-erosion rate cycles, displaying >10-fold rate changes (~0.02 to 0.23 mm/yr) that are largely in-phase with predicted Milankovitch long eccentricity cycles at the 400-kyr frequency (Fig. 4 to Fig. 6), a correlation that is discussed in detail below.

### Paleo-erosion Rates from 2.4 Ma to 1.8 Ma

The final period of the erosion-rate record from ~2.4 to 1.8 Ma departs from the 400-kyr cyclicity and is characterized instead by a large increase in erosion rates, which fluctuate about a mean consistent with the modern rate of ~0.34 mm/yr (Fig. 5).

### CLIMBER-2 Climate Model

**Fig. 7:**
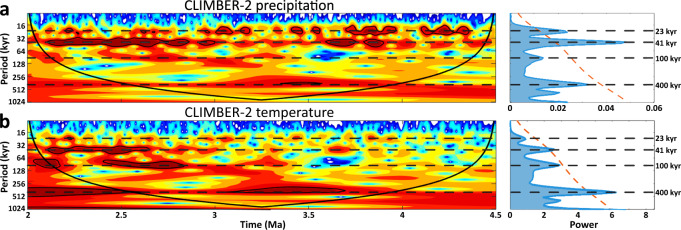
CLIMBER-2 wavelet and power spectra results. **a** CLIMBER-2 precipitation and **b** temperature wavelet analyses and power spectra. The grid cell of CLIMBER-2 covers the area from 30–20°S and 65–116.4°W. Precipitation shows strong 23-kyr periodicities from 4.5 to 3 Ma, with strong 41-kyr periodicities from 3 to 2 Ma. In contrast, the temperature spectra are largely dominated by 400-kyr periodicities throughout both periods with 100-kyr and 41-kyr periodicities increasing in power at <3 Ma. The continuous black line in the wavelet analysis indicates areas of the spectrum susceptible to distortion due to edge effects of the time series. The dashed red line in the power spectrum and the black outlined areas in the wavelet plots indicate the 95%-confidence interval. Horizontal dashed lines in the power spectrum indicate the dominant orbital frequencies at 23, 41, 100, and 400-kyr periodicities.

Results from the CLIMBER-2 model suggest that, although precipitation oscillations in the region are dominated by the 23-kyr precession and 41-kyr obliquity-frequency bands during this period (Fig. 7), 400-kyr eccentricity cycles strongly modulate changes in amplitude of the 23-kyr precipitation cycles (Fig. 4e, f). When eccentricity is high, for example, precession cycles cause ~20% swings in summer insolation at 23°S, cycles that are damped to ~2% during periods of low eccentricity (Fig. 4b). These model results are notable because paleo-erosion rates correlate with the amplitude of CLIMBER-2 precipitation oscillations from ~4.0 to 2.4 Ma (Fig. 6), which themselves are largely driven by insolation.

Although spatially coarse, several lines of evidence suggest that the CLIMBER-2 data are, broadly, a reasonable predictor of Plio-Pleistocene climatic variability. For example, strong 400-kyr Milankovitch pacing is observed in Antarctic ice volume, sea surface temperature, and global carbon-cycle dynamics during the Pliocene^51,52^. Likewise, the dominance of the 23-kyr frequency oscillation in the CLIMBER-2 precipitation results is in agreement with late Quaternary hydrologic records across South America linking SAMS precipitation intensity to precessionally-driven summer-insolation maxima^53,54^. Finally, the Plio-Pleistocene CLIMBER-2 model results mimic modern north-south hemispheric precipitation gradients, indicating adequate sensitivity to latitudinal moisture transport in the model (Fig. 1b and Supplementary Fig. 5).

## Discussion

**Fig. 8:**
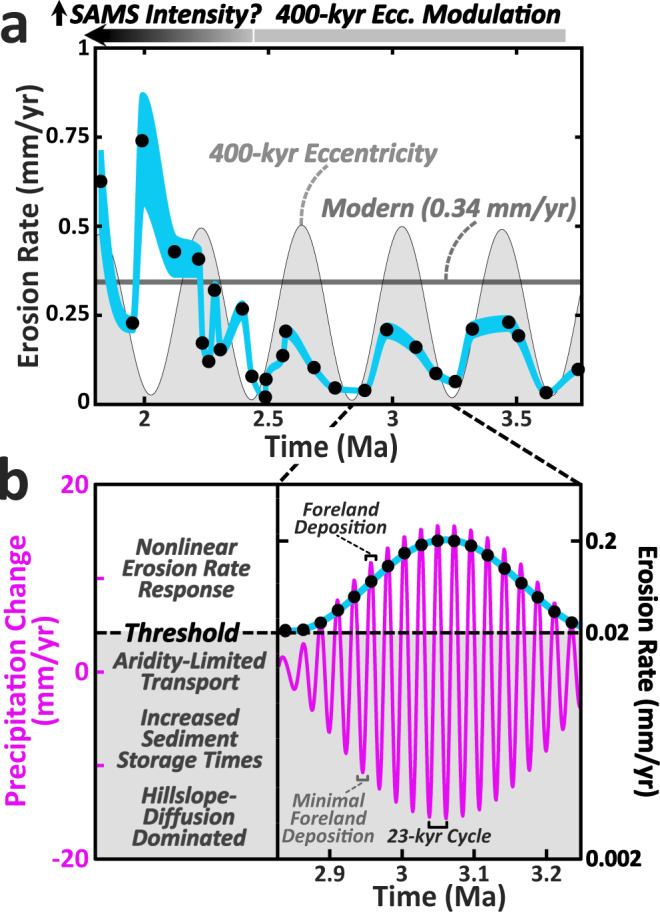
Paleo-erosion rates and conceptual mechanism for observed 400-kyr cycles. **a** Calculated ^10^Be-derived paleo-erosion rates (±2*σ* envelope) from 1.8-3.8 Ma compared to modern ^10^Be-derived erosion rates (0.34 ± 0.01 mm/yr) and 400-kyr eccentricity pacing. Erosion-rate values shown are based on the preferred uplift scenario (Supplementary Fig. 4b). **b** Conceptual model illustrating how a 400-kyr erosion-rate signal with an order-of-magnitude variation in erosion rates might be produced from a nonlinear erosional response to precession-forced precipitation variability (in purple; Fig. 4f) above a hydrologic threshold for transport/landsliding. Black dots represent erosion rates that track the precipitation amplitude above the threshold (dashed black line) for each 23-kyr precession cycle. Note the nonlinear erosion-rate axis. SAMS = South American Monsoon System.

The most striking result of this study is the correlation between erosion rate and the 400-kyr amplitude modulation of 23-kyr summer insolation oscillations from ~4.0 to 2.4 Ma (Fig. 5a, Fig. 6b). Given the synchrony of observed erosion-rate cycles with the amplitude of precipitation changes predicted by the CLIMBER-2 model (Fig. 4b–d and Fig. 8a), we hypothesize that changes in erosion rates during this period are driven by changes in summer monsoon precipitation in the study area. Although we argue for precipitation changes as the primary driver of erosion rates, the preservation of orbitally-paced erosion-rate cyclicity during this limited time window (~4-2.4 Ma) was made possible by uniquely favorable tectonic and geomorphic conditions within the watershed. For example, the onset of the clear cyclicity at ~4.0 Ma corresponds to a major shift in sediment provenance described above, which is interpreted to record a more restricted sediment source, and the possible addition of sediments from the interior margin of the Puna Plateau^32^(Supplementary Fig. 3).

Although the observed orbital pacing of erosion rates suggests a direct climatic control on long-term erosion rates, any model to explain this observed correlation must answer several key questions. First, given that erosion rates are generally rapid enough to record changes at 23-kyr precessional timescales, why is this signal, or noise associated with undersampling it, not observed? Relatedly, if such a high-frequency signal is simply being ‘smoothed’ by the geomorphic system, why then should erosion rates rise and fall in 400-kyr cycles when modeled precipitation is essentially constant when averaged over multiple 23-kyr precessional cycles? Finally, how do we reconcile order-of-magnitude changes in observed erosion rates with apparently modest changes in modeled mean annual precipitation (~20%) during this period (Fig. 1b)?

Recognizing these questions, our preferred hypothesis to explain the observed 400-kyr erosion-rate pattern calls upon nonlinear threshold hillslope behavior driven by landsliding coupled to channel incision (e.g., lateral or vertical)^55,56^, causing erosion rates to nonlinearly track precipitation amplitudes above some threshold (Fig. 8)^57^. During periods of increased aridity, sediment production and transport would diminish significantly, thereby allowing long-timescale, slower hillslope diffusion processes to dominate the erosion-rate signal. During wetter periods, erosion rates would be dominated by both an increased frequency of landsliding and greater exceedance of transport and incision thresholds, volumetrically overwhelming and diluting cosmogenic radionuclide signals generated during preceding transport-limited periods: similar to model predictions of punctuated denudation driven by increasing runoff intensity^2^. Although steady-state exploration of stochastic stream power models in the study area during the Pliocene is beyond the scope of this study, previous work indicates that climatic perturbations are most likely to influence erosion rates in semiarid landscapes characterized by steep channels, highly variable flood discharges, and sustained flood variability with increasing mean annual runoff^58^. These characteristics as well as the importance of landsliding frequency and landscape adjustment in modulating sediment flux in the study region is well documented and has been previously implicated in explaining order-of-magnitude, erosion-rate variability observed for millennial-scale wet-arid climate transitions during the late Quaternary^13,57,59^. In addition, the magnitude of paleo-erosion rates calculated in this study agrees with empirical landscape thresholds documented in the study region, whereby mean catchment specific stream power values become asymptotic with increasing catchment erosion rates between 0.1–0.5 mm/yr^57^, strongly implicating landslide dynamics as a primary driver of erosion rates in this landscape. The preferred hypothesis is also indirectly supported by results from higher-resolution, regional paleoclimate modeling in the study area that predicts greater interannual climatic variability along the eastern Andes during the Pliocene compared to more recent time periods (~21 ka, 6 ka, pre-industrial)^60^, providing a potential climatic mechanism for punctuated exceedance of erosional thresholds and sustained runoff variability in the Río Iruya region.

Whereas our hypothesized mechanism provides a compelling explanation that satisfies the observed results and is well-documented in the modern study region, other potential explanations are certainly possible. For example, the simplest explanation for the observed 400-kyr pacing of erosion rates is that Pliocene moisture transport into the study area was driven directly by 400-kyr eccentricity cycles instead of modulating precession amplitudes. Although evidence exists for strong pacing by long-eccentricity cycles in select marine sea-surface temperature and carbon-isotope records during the Pliocene^52^, such a scenario contradicts the precession-dominated CLIMBER-2 precipitation results during this time (Fig. 4 and Fig. 7). This explanation also contradicts empirical evidence from late-Quaternary continental records in the Andes documenting the dominance of higher-frequency precipitation oscillations largely driven by precession and/or 100-kyr eccentricity cycles^53,54^, though we acknowledge the potential disconnect between these geologically recent records and the climate state of the Pliocene.

Alternatively, the 400-kyr cycles driving erosion rates may derive from temperature oscillations instead of precipitation. The 400-kyr frequency dominates the CLIMBER-2 temperature signal during the Pliocene (Fig. 7b) and the effect of temperature oscillations would have been especially pronounced if large glaciers and/or periglacial processes were at work in the catchment during this period^23,61^. However, there is sparse evidence to suggest that the Río Iruya catchment was glaciated even during the Last Glacial Maximum (LGM) (~20 ka), with most regional glaciers during the LGM small and localized to only the highest elevations (> 4000 m)^62^. In addition, modern mean Austral winter temperatures at ~2800 m elevation in the headwaters of the study area currently average ~7–8 °C with average low temperatures of ~0–1 °C (town of Iruya). The Pliocene is known to have had a mean global temperature similar, if not slightly warmer, to present day^63^, making it unlikely that periglacial/glacial processes were responsible for the observed erosion rate variability during the period of study.

Another possible mechanism for the observed erosion-rate cyclicity is that catchment erosion rates stayed relatively constant throughout the period from 4–2.4 Ma and that the observed signal arises from variations in sediment-transport dynamics that impacted ^10^Be concentrations, namely sediment provenance concentrations and/or stochastic burial during transport. One such scenario is that the onset of cyclicity at 4 Ma was triggered by the Iruya headwaters tapping into a previously restricted basin(s) on the eastern Puna Plateau and rapidly evacuating its sedimentary fill into the foreland. Cyclic changes in monsoonal strength could then extract pulses of low-concentration sediments into the foreland (e.g., the Oran group) during high-amplitude cycles, giving the impression of faster erosion. Alternatively, cycles of enhanced fluvial storage at shallow burial depths would allow additional ^10^Be to build up during the transport process. Cyclic reworking and final burial of these sediments could create periods of apparently slower erosion. These explanations appear unlikely based on results from end-member modeling for both scenarios (Supplementary Fig. 6). Periods of apparently rapid erosion would require roughly 50–90% of sediment be derived from a source with zero ^10^Be concentration to produce the observed cyclicity in an otherwise steadily eroding landscape. Likewise, periods of apparently slow erosion rates would require shallow sediment storage (<1 m depth) over timescales of 10–30 kyr, which seems unlikely given the proximity of the relatively steep Río Iruya watershed to the depositional loci and the general lack of accommodation space in the modern watershed (e.g., narrow canyons and lack of paleo-terraces in all but the highest reaches) (Supplementary Fig. 7). Finally, cyclic tapping of long-buried sediments seems unlikely given that ^26^Al/^10^Be ratios are mostly within uncertainty of their expected production rate ratios (Fig. 2C).

A fourth potential explanation is that the 400-kyr erosion-rate signal is the product of a climatically driven 23-kyr erosion-rate cycle that has been filtered by geomorphic processes within the watershed. For example, varying timescales of sediment transport and storage could act to overprint the underlying hillslope erosion rate. Much of our current understanding of how source watersheds respond to and convey climate signals to depositional areas relies on numerical models with conflicting arguments that include shredding, dampening, and even amplification of sediment-flux signals by autogenic geomorphic processes^64,65^. Empirical results from nearby paleo-erosion rate studies^13,19^ have proposed that catchment architecture and transport distance may dictate which periodicities are preserved in the sediment record, whereby preserved periodicities are proportional to the square of the river length above the site of deposition^19^. Whereas this regional observation is compelling, the Río Iruya dataset represents an integrated geochemical signature of catchment erosion that is largely independent from the autogenic geomorphic processes that drive fluvial terrace or alluvial fan preservation. Evidence from experimental studies, however, indicates that catchments undergoing pronounced aggradation/incision cycles, and when coupled to significant long-term sediment storage, may result in mixing temporally disparate erosion-rate signals during periods of incision as modern ^10^Be signals are progressively mixed with stratigraphically older, cosmogenically-decayed signals^66^. Although some temporal mixing of cosmogenic radionuclide signals by geomorphic processes is necessary to allow precession amplitudes to closely track 400-kyr cycles based on our hypothesized mechanism, the storage times necessary to decay a shielded ^10^Be signal to produce the observed 400-kyr cyclicity in our record would be prohibitive (»10^6^ years based on ^10^Be half-lives).

In summary, we posit that, during the Pliocene, precipitation was controlled by the strength of the SAMS, which oscillated on a 23-kyr timescale at an amplitude controlled by longer 400-kyr eccentricity cycles. The Río Iruya watershed, favorably conditioned by tectonically steepened and likely poorly vegetated threshold hillslopes (similar to the modern catchment), responded nonlinearly via landslide dynamics to this precipitation regime, allowing relatively small perturbations in precipitation (±20%) to produce order-of-magnitude changes in erosion rates in concert with 400-kyr pacing (Fig. 8).

Another interesting feature of the paleo-erosion rate dataset is the overall increase in erosion rate and loss of the 400-kyr cyclicity starting at ~2.4 Ma (Fig. 5a). One possible explanation is that the loss of 400 kyr erosion-rate cyclicity may have been driven by a watershed reorganization in the headwaters of the Río Iruya associated with the ~2.4 Ma provenance shift. Alternatively, the loss of erosion-rate cyclicity may have resulted from a change in precipitation conditions associated with the continued growth of the Eastern Cordillera, eventually concentrating precipitation along the eastern range front as the orographic barrier became more pronounced. This explanation is supported during this time by an order-of-magnitude decrease in erosion rates on the leeward side of the growing Eastern Cordillera in the Quebrada de Humahuaca^21^ (Fig. 5 and Supplementary Fig. 3). The ongoing tectonism and increased precipitation on the windward flank of the Eastern Cordillera, when combined with a potentially shorter, steeper catchment would have conspired to drive the more rapid erosion rates observed following the Plio-Pleistocene transition. However, it remains unclear why the newly reorganized catchment would not continue to record precipitation cyclicity during this period, given the relatively faithful tracking of 400-kyr cyclicity over the preceding ~1.5 Myr period (Fig. 5a).

A third possible explanation for the loss of erosion-rate cyclicity at ~2.4 Ma is a change in the global climate state driven by the onset of major Northern Hemisphere glaciation at the Plio-Pleistocene boundary around 2.6 Ma. Growth of the Northern Hemisphere ice sheets ushered in a climate regime largely paced by obliquity (~41 kyr) as the earth progressively cooled from the Pliocene into the Pleistocene:^51,67^ a finding supported by the CLIMBER-2 precipitation results as well (Fig. 4f and Fig. 7). The CLIMBER-2 data also suggest that, as obliquity begins to dominate the precipitation signal between 3–2.5 Ma, the amplitude becomes modulated by eccentricity mega-cycles (~1.2 Myr), producing both higher amplitude oscillations than the precession cycles and decreased variability between individual oscillations due to the protracted length of the modulating cycle (Fig. 4f). If erosion rates continued to track amplitude modulations of the dominant obliquity signal instead of precession during this time, it is plausible that the more gradual changes in amplitude associated with the eccentricity mega-cycles failed, or were too subtle, to be recorded by erosion rates within the catchment. Alternatively, the strengthening of the obliquity signal in combination with a diminishing precession signal during the early Pleistocene may have produced a more chaotic temporal precipitation regime that, when coupled to a newly evolving basin architecture, prevented any dominant climate forcing frequency to emerge in the erosion-rate data. More broadly, the SAMS may also have undergone significant changes during this period as El Niño Southern Oscillation conditions supplanted those of the permanent El Niño state^68,69^ and the Intertropical Convergence Zone was pushed southward due to increased interhemispheric temperature gradients driven by Northern Hemisphere ice-sheet growth^70^.

## Methods

### Sample age model and uncertainty

Detailed timing in the Río Iruya section was established using a densely sampled magnetostratigraphic transect combined with several precisely dated, intercalated volcanic ash beds yielding robust age control in the section that spans 1.94 to 6.49 Ma^32^ (Supplementary Fig. 2 and Supplementary Dataset 1). Calculation of age uncertainties on paleo-erosion rate samples was performed using published magnetostratigraphic data and allowed the polarity reversal boundaries to be flexible with respect to their confining samples or radiometric ash ages. Assuming a linear accumulation rate between polarity reversals, the maximum and minimum age of each cosmogenic radionuclide sample was determined by moving the reversal boundaries as far forward (making the sample older) and as far back (making the sample younger) as possible in the stratigraphy (Supplementary Fig. 8). This method allows for proper quantification of sample-age uncertainty and highlights the asymmetric nature of age uncertainty associated with unevenly spaced sampling and polarity reversal boundaries. Midpoint, maximum, and minimum age uncertainties for each sample are reported in Supplementary Dataset 2, with a median maximum and minimum divergence from the midpoint age of 0.033 Myr (*n* = 41) and 0.034 Myr (*n* = 39), respectively. For samples in the lower part of the section defined only by ashes (5.5–6.49 Ma), the midpoint age was calculated using a linear interpolation based on stratigraphic location between the ashes. Uncertainties for these samples were cautiously set as the 75th percentile of maximum and minimum uncertainties from the magnetostratigraphy component of the dataset (0.048 and 0.049 Ma, respectively). In the case of the youngest sample (IR22c), which lacked lower bound age control, the midpoint age was calculated using the average sedimentation rate for the entire section (930 m/Ma), yielding an age of 1.83 Ma with uncertainties again assumed to be within the overall 75th percentile. In cases where a maximum age uncertainty could be defined but not a minimum one (IR20.1c and IR21c), due to the topmost reversal boundary lying down section from the cosmogenic radionuclide sample, we assumed uncertainty symmetry about the midpoint and used the maximum uncertainty value for both uncertainties.

### Cosmogenic radionuclide sampling strategy and analytical procedures

Quartz-rich, cobble conglomerate beds ~1-m thick were excavated in the Oran Group section along the Río Iruya and sieved in the field to obtain the <1 mm interstitial size fraction. Samples were then further sieved at Middlebury College to obtain the 0.25- to 1-mm fraction. This fraction then followed a standard quartz cleaning protocol^71^ whereby samples were subjected to carbonate dissolution using a heated 1:1 hydrochloric acid mixture and repeated 2% hydrofluoric acid leachings performed using commercial hot dog rollers and ultrasonic baths to provide heat and mechanical abrasion. Sample purities were checked following three rounds of 2% hydrofluoric acid leaching and continued until quartz purity targets were met. Quartz dissolution (ranging from 95 to 621 g per sample) using hydrofluoric acid, ^10^Be and ^26^Al extraction by ion-exchange chromatography, and sample-target preparation were all performed at the University of California at Santa Barbara using established and publicly available protocols^57,71^. During digestion of the quartz samples, a low-ratio ^9^Be spike with a ^10^Be/^9^Be ratio value of 3 × 10^−15^ was added. In addition, during each digestion batch an empty teflon beaker was treated with approximately the same maximum volume of hydrofluoric acid as used in that given batch of samples and was spiked with the same low-ratio ^9^Be spike. These blanks were then processed through the same protocols as samples, yielding ^10^Be/^9^Be values for the 5 blanks ranging from 3.5 to 13 × 10^−15^ (Supplementary Dataset 2 and 3). No ^27^Al spike was added, given the high concentrations of Al in the quartz mineral grain lattice, with no ^26^Al contamination detected in the processed blanks during the chemical processing steps. ^10^Be and ^26^Al targets were then measured via accelerator mass spectrometry at the Purdue Rare Isotope Measurement Laboratory (PRIME) using the 07KNSTD standardization and assuming a ^10^Be half-life of 1.36 ± 0.07 Myr^72^. Reference standard KNSTD was used for ^26^Al assuming a half-life of 0.705 Myr^30^. In total, 49 ^10^Be and 23 ^26^Al samples were processed and analyzed with ^26^Al measurements restricted to samples with ages <3.5 Ma (~5 ^26^Al half-lives).

### Modern erosion rates from ^10^Be

Modern erosion rates were calculated using ^10^Be for catchment samples taken ~3 km upstream of the sampled section of the Río Iruya canyon (IR-3M) as well as further upstream in the headwaters of the Río Iruya (IR-6M) (Supplementary Fig. 3). The production of ^10^Be in quartz in the upper few meters of the Earth’s surface arises predominantly from high-energy neutron bombardment of O and Si nuclei, with negative muon capture and fast muon reactions dominant at depths greater than ~3 meters^31,73,74^. Production *P* of ^10^Be can be modeled as an exponential decay function with depth from the surface for each production pathway where

1 $$P={P}_{0}{e}^{-z\rho /\varLambda }$$

and *P*_*0*_ (at g^−1^ yr^−1^) is the surface production rate, z (cm) is the depth below the surface, *ρ* the density of the regolith (g cm^−3^), and $\varLambda$ the attenuation mean free path (g cm^−2^)^31^. Because *P*_*0*_ varies with latitude, altitude, and topographic shielding, modern mean effective production rates (topographic shielding factor x production rate) across the Río Iruya watershed were estimated using the 90-meter Shuttle Radar Topography Mission digital elevation model and the CRONUScalc Matlab codes^75^ assuming a sea-level high-latitude (SLHL) production rate of 4.01 atoms g^−1^ yr^−1^ and based on the time-independent Lal-Stone scaling (St)^31,76,77^. Assuming rapid transport of sediments from the hillslopes into the fluvial network and proper amalgamation in the fluvial system of an integrated catchment signal, the concentration of ^10^Be in a river sand sample can be used to calculate the erosion rate, R_E_, of the upstream catchment^24^ where

2 $${R}_{E}=\frac{\varLambda }{\rho }\left(\frac{{P}_{0}}{{N}_{E}}-\lambda \right)$$

and $\lambda$ is the radioactive decay constant for ^10^Be (yr^−1^) and N_E_ is the ^10^Be concentration of a modern river sample (atoms g^−1^).

### Paleo-erosion rate calculations and uncertainty propagation

Paleo-erosion rate calculations were performed using a simplified version of the detailed methodology presented in^17^, which tracks and compounds uncertainties across the different cosmogenic radionuclide ingrowth components. Here we omit any treatment of the excavated depth of sampling or paleochannel thickness, due to the rapid exhumation rates (~1 m/yr for 150 years) in the Río Iruya and the lack of paleochannel-derived samples that might warrant such nuanced treatment. The major ingrowth and decay components of the measured cosmogenic radionuclide signal (N_A_) are modeled by the linear combination:

3 $${{{{{{\rm{N}}}}}}}_{{{{{{\rm{A}}}}}}}{={{{{{\rm{N}}}}}}}_{{{{{{\rm{E}}}}}}}{+{{{{{\rm{N}}}}}}}_{{{{{{\rm{T}}}}}}}{+{{{{{\rm{N}}}}}}}_{{{{{{\rm{B}}}}}}}{{-}{{{{{\rm{N}}}}}}}_{{{{{{\rm{D}}}}}}}{+{{{{{\rm{N}}}}}}}_{{{{{{\rm{X}}}}}}}$$

where N_A_ is the total measured concentration of each sample, which encompasses the contribution from erosion in the watershed (N_E_ – the value of interest), transport to the site of deposition (N_T_), spallation-induced and muogenic production during burial (N_B_), radioactive decay during burial (N_D_), and lastly, exhumation to the surface where the sediment is re-exposed to cosmogenic bombardment (N_X_) and then sampled^17^. Production from spallation and fast and slow muons is integrated throughout both the burial and exhumation terms (cf. Supplementary Table 1), though production during exhumation in this study was negligible due to the rapid incision of the canyon. Each component and associated uncertainty were constrained and inverted progressively back through time (N_X_ to N_E_) utilizing a Monte Carlo approach (100,000 iterations) to compound uncertainty in the erosion-rate component (N_E_) and calculated erosion rate (R_E_) for each sample based on Eq. 2 (Supplementary Dataset 2 and 3 and Supplementary Table 1). Resulting asymmetric uncertainty distributions derive from the exponential depth dependence of cosmogenic radionuclide production, the time dependence of decay, and asymmetry in age uncertainties (Supplementary Fig. 8). Whereas uncertainties were not assigned to production rate values in the source area (P_E_) and foreland depocenter (P_B_), several scenarios of production-rate evolution through time were modeled to account for a wide range of possible catchment uplift scenarios (see Supplementary Fig. 4 and Supplementary Dataset 2). These differing scenarios, it should be noted, only result in minor changes to the absolute magnitude of calculated paleo-erosion rates from 1.8 Ma to 4 Ma and not the observed patterns or cyclicity. The transport contribution (N_T_) to measured cosmogenic radionuclide concentrations in the analysis is assumed to be negligible based on: (1) the close proximity of the depositional basin to a relatively steep source area starting at ~4 Ma;^32,44^ and (2) good agreement between ^26^Al:^10^Be sample ratios (for ^26^Al AMS errors <35%) with expected ^26^Al:^10^Be ratios assuming no significant burial during transport from ~2–3 Ma (Fig. 2c). Our calculations assume a relative contribution by nucleon spallation, negative muon capture, and fast muon reactions at the surface equal to 98.15%, 1.2%, and 0.65%, respectively^73,74,78^. To calculate the decay of ^10^Be during burial, a half-life of 1.387 Myr^29^ was used as compared to the half-life of 1.36 Myr^72^ assumed during analytical measurement. All additional calculated, assumed, and measured parameters are detailed in Supplementary Table 1. Note that no attempt was made to derive paleo-erosion rates from ^26^Al due to the larger analytical uncertainties resulting from the dilution of the ^26^Al/^27^Al ratio by abundant ^27^Al quartz impurities in the sample material. A detailed presentation and discussion of the uncertainty workflow and equations used in the paleo-erosion rate analysis, as well as open-source R code, is available in^17^.

### CLIMBER-2 climate model simulations and data

The Earth system model of intermediate complexity, (EMIC) CLIMBER-2, was chosen to test the hypothesis that climate perturbations in the Río Iruya region largely result from global-scale forcings during the Plio-Pleistocene. The low-resolution, intermediate complexity model seeks to incorporate a large set of processes and feedbacks normally reserved for comprehensive climate models, but at a low enough spatial resolution to avoid their computational costs, thereby yielding fast turnaround times for long time-scale paleoclimate reconstructions. Whereas the low-spatial resolution of the model is incapable of resolving orographic and vegetative forcings associated with the Andes (and thereby associated regional systems like the South American low level jet), CLIMBER-2 has been shown to reliably replicate both large-scale climate reconstructions from the Last Glacial Maximum, as well as more complex model simulations for the Pliocene^79,80^.

Here we implemented version 2.3 of CLIMBER-2 that includes atmospheric, ocean, and terrestrial components, including a dynamic vegetation model and thermodynamic sea-ice model^50,79^. The CLIMBER-2 atmospheric component is represented by a 2.5-dimensional statistical-dynamical model with latitudinal resolution of 10° and longitudinal resolution of ~51° (7 zones) using a daily time step. Circulation, temperature, and humidity are calculated across 10 vertical levels in the model and 16 levels for long-wave radiation. Vegetation potential in each grid cell is recorded at a yearly time-step based on temperature and precipitation using the terrestrial model VECODE^81^. The ocean model integrates the Pacific, Atlantic, and Indian oceans via connection through the Southern Ocean and calculates zonally averaged flow between grid cells, as well as temperature and salinity changes, at a time step of 5 days, a latitudinal resolution of 2.5°, and across 20 unevenly distributed vertical levels^82^.

Model simulations were performed using a transient 5-million-year simulation which utilizes an established solution for astronomical parameters^83^ to calculate insolation, along with prescribed changes of ice-sheet topography and atmospheric CO_2_ concentrations^79^. Ice-sheet topography change is adopted from previous simulations of 3-D ice-sheet models^52^ driven by the LR04 benthic δ18O stack record^67^. Temperature derived from the simulation with the 3-D ice-sheet model is used to reconstruct CO_2_ concentration using previously described methods^84^. Whereas precipitation and temperature are recorded daily for each cell in the model, outputs in this paper were derived from 1000-year time-step averages to filter out sub-millennial model variability not pertinent to orbital time scales. Frequency analysis was performed using AnalySeries software version 2.0.8. Both precession and long-eccentricity frequency filtering components were acquired using Gaussian shaped filters at 0.044 and 0.0025 with a bandwidth of 0.008 and 0.001, respectively. Wavelet analyses were performed using previously described methods^85^. Due to the simple spatial representation of landmass in the CLIMBER-2 model, a geographic offset was accounted for in assessing data for South America (e.g., Fig. 1b) as the continent is shifted westward by ~35–45 degrees of longitude in the model (see Supplementary Fig. 5). CLIMBER-2 outputs used in this paper are provided in Supplementary Dataset 4.

## Supplementary information

Supplementary Information

Description of Additional Supplementary Files

Supplementary Dataset 1

Supplementary Dataset 2

Supplementary Dataset 3

Supplementary Dataset 4

## Data Availability

All cosmogenic radionuclide and CLIMBER-2 data generated in this study are provided in the Supplementary Information.

## Code availability

All computational analyses rely on previously published models and code directly cited within the methods of the manuscript.

## References

[1] Gilbert GK (1900). Rhythms and geologic time. Science.

[2] Tucker GE, Slingerland R (1997). Drainage basin responses to climate change. Water Resour. Res..

[3] Willett SD (1999). Orogeny and orography: The effects of erosion on the structure of mountain belts. J. Geophys. Res.-Solid Earth.

[4] Godard V, Tucker GE, Burch Fisher G, Burbank DW, Bookhagen B (2013). Frequency-dependent landscape response to climatic forcing. Geophys. Res. Lett..

[5] Braun J, Voisin C, Gourlan AT, Chauvel C (2015). Erosional response of an actively uplifting mountain belt to cyclic rainfall variations. Earth Surf. Dyn..

[6] Whipple KX (2009). The influence of climate on the tectonic evolution of mountain belts. Nat. Geosci..

[7] Bookhagen, B. & Strecker, M. R. Orographic barriers, high-resolution TRMM rainfall, and relief variations along the eastern Andes. *Geophys. Res. Lett.* **35**, L06403, 10.1029/2007GL032011 (2008).

[8] Willenbring JK, von Blanckenburg F (2010). Long-term stability of global erosion rates and weathering during late-Cenozoic cooling. Nature.

[9] Molnar P (2004). Late Cenozoic increase in accumulation rates of terrestrial sediment: how might climate change have affected erosion rates?. Annu. Rev. Earth Planet. Sci..

[10] Schaller M (2004). Paleoerosion rates from cosmogenic 10Be in a 1.3 Ma terrace sequence: Response of the River Meuse to changes in climate and rock uplift. J. Geol..

[11] Charreau J (2011). Paleo-erosion rates in Central Asia since 9Ma: A transient increase at the onset of Quaternary glaciations?. Earth Planet. Sci. Lett..

[12] Hidy AJ, Gosse JC, Blum MD, Gibling MR (2014). Glacial–interglacial variation in denudation rates from interior Texas, USA, established with cosmogenic nuclides. Earth Planet. Sci. Lett..

[13] Schildgen TF (2016). Landscape response to late Pleistocene climate change in NW Argentina: Sediment flux modulated by basin geometry and connectivity. J. Geophys. Res.: Earth Surf..

[14] Val P, Hoke GD, Fosdick JC, Wittmann H (2016). Reconciling tectonic shortening, sedimentation and spatial patterns of erosion from 10Be paleo-erosion rates in the Argentine Precordillera. Earth Planet. Sci. Lett..

[15] Marshall JA, Roering JJ, Gavin DG, Granger DE (2017). Late Quaternary climatic controls on erosion rates and geomorphic processes in western Oregon, USA. Geol. Soc. Am. Bull..

[16] Puchol N (2017). Limited impact of Quaternary glaciations on denudation rates in Central Asia. Geol. Soc. Am. Bull..

[17] Oskin ME (2017). Steady 10 Be-derived paleoerosion rates across the Plio-Pleistocene climate transition, Fish Creek-Vallecito basin, California. J. Geophys. Res.: Earth Surf..

[18] Amidon WH (2017). Mio-Pliocene aridity in the south-central Andes associated with Southern Hemisphere cold periods. Proc. Natl Acad. Sci..

[19] Tofelde S (2017). 100 kyr fluvial cut-and-fill terrace cycles since the Middle Pleistocene in the southern Central Andes, NW Argentina. Earth Planet. Sci. Lett..

[20] Madella A, Delunel R, Akçar N, Schlunegger F, Christl M (2018). ^10^Be-inferred paleo-denudation rates imply that the mid-Miocene western central Andes eroded as slowly as today. Sci. Rep..

[21] Pingel H, Schildgen T, Strecker MR, Wittmann H (2019). Pliocene–Pleistocene orographic control on denudation in northwest Argentina. Geology.

[22] Lenard SJP (2020). Steady erosion rates in the Himalayas through late Cenozoic climatic changes. Nat. Geosci..

[23] Mariotti A (2021). Nonlinear forcing of climate on mountain denudation during glaciations. Nat. Geosci..

[24] Charreau J (2021). A 6 Ma record of palaeodenudation in the central Himalayas from in situ cosmogenic 10 Be in the Surai section. Basin Res..

[25] Mandal, S. K., Scherler, D. & Wittmann, H. Tectonic accretion controls erosional cyclicity in the Himalaya. *AGU Adv.* **2**, e2021AV000487 (2021).

[26] Rohrmann, A. et al. Miocene orographic uplift forces rapid hydrological change in the southern central Andes. *Sci. Rep.* **6**, 35678, 10.1038/srep35678 (2016). 10.1038/srep35678 PMC5073360 27767043

[27] Hoorn C (2010). Amazonia through time: andean uplift, climate change, landscape evolution, and biodiversity. Science.

[28] von Blanckenburg F (2006). The control mechanisms of erosion and weathering at basin scale from cosmogenic nuclides in river sediment. Earth Planet. Sci. Lett..

[29] Chmeleff J, von Blanckenburg F, Kossert K, Jakob D (2010). Determination of the 10Be half-life by multicollector ICP-MS and liquid scintillation counting. Nucl. Inst. Methods Phys. Res., B.

[30] Nishiizumi K (2004). Preparation of 26Al AMS standards. Nucl. Instrum. Methods Phys. Res. Sect. B: Beam Interact. Mater. At..

[31] Lal D, Labeling Cosmic-Ray (1991). of Erosion Surfaces—Insitu nuclide production-rates and erosion models. Earth Planet. Sci. Lett..

[32] Amidon WH (2015). Provenance and tectonic implications of Orán Group foreland basin sediments, Río Iruya canyon, NW Argentina (23° S). Basin Res..

[33] Echavarria L, Hernández R, Allmendinger R, Reynolds J (2003). Subandean thrust and fold belt of northwestern Argentina: Geometry and timing of the Andean evolution. AAPG Bull..

[34] Hernández RM, Reynolds J, Di A (1996). Salvo, Análisis tectosedimentario y ubicación geocronológica del Grupo Orán en el río Iruya. Boletín de. Informaciones Petroleras.

[35] Starck D, Schulz A (1996). La configuración estructural del límite Cordillera Oriental – Sierras Subandinas en el extremo norte de la República Argentina. XII Congr. Geol.ógico Boliviano, Mem..

[36] Hongn F (2007). Middle Eocene deformation and sedimentation in the Puna-Eastern Cordillera transition (23°−26°S): Control by preexisting heterogeneities on the pattern of initial Andean shortening. Geology.

[37] Insel N (2012). Paleozoic to early Cenozoic cooling and exhumation of the basement underlying the eastern Puna plateau margin prior to plateau growth. Tectonics.

[38] Kleinert K, Strecker MR (2001). Climate change in response to orographic barrier uplift: Paleosol and stable isotope evidence from the late Neogene Santa Maria basin, northwestern Argentina. Geol. Soc. Am. Bull..

[39] Mulch A, Uba CE, Strecker MR, Schoenberg R, Chamberlain CP (2010). Late Miocene climate variability and surface elevation in the central Andes. Earth Planet. Sci. Lett..

[40] DeCelles PG, Carrapa B, Horton BK, Gehrels GE (2011). Cenozoic foreland basin system in the central Andes of northwestern Argentina: Implications for Andean geodynamics and modes of deformation. Tectonics.

[41] Reynolds JH (2000). Middle Miocene tectonic development of the transition zone, Salta Province, northwest Argentina: Magnetic stratigraphy from the Metan Subgroup, Sierra de Gonzalez. Geol. Soc. Am. Bull..

[42] Reynolds JH, Hernández RM, Galli CI, Idleman BD (2001). Magnetostratigraphy of the Quebrada La Porcelana section, Sierra de Ramos, Salta Province, Argentina: age limits for the neogene Orán Group and uplift of the southern Sierras Subandinas. J. South Am. Earth Sci..

[43] Rahl JM, Harbor DJ, Galli CI, O’Sullivan P (2018). Foreland basin record of uplift and exhumation of the Eastern Cordillera, Northwest Argentina. Tectonics.

[44] Pingel H, Strecker MR, Alonso RN, Schmitt AK (2013). Neotectonic basin and landscape evolution in the Eastern Cordillera of NW Argentina, Humahuaca Basin (~24°S). Basin Res..

[45] Streit RL (2015). Controls on intermontane basin filling, isolation and incision on the margin of the Puna Plateau, NW Argentina (~23°S). Basin Res..

[46] Strecker MR (2007). Tectonics and climate of the Southern Central Andes. Annu. Rev. Earth Planet. Sci..

[47] Vuille M (2012). A review of the South American monsoon history as recorded in stable isotopic proxies over the past two millennia. Climate.

[48] Garreaud RD, Molina A, Farias M (2010). Andean uplift, ocean cooling and Atacama hyperaridity: A climate modeling perspective. Earth Planet. Sci. Lett..

[49] Uba CE, Strecker MR, Schmitt AK (2007). Increased sediment accumulation rates and climatic forcing in the central Andes during the late Miocene. Geology.

[50] Petoukhov V (2000). CLIMBER-2: a climate system model of intermediate complexity. Part I: model description and performance for present climate. Clim. Dyn..

[51] Zachos J, Pagani M, Sloan L, Thomas E, Billups K (2001). Trends, rhythms, and aberrations in global climate 65 Ma to present. Science.

[52] de Boer B, Lourens LJ, van de Wal RSW (2014). Persistent 400,000-year variability of Antarctic ice volume and the carbon cycle is revealed throughout the Plio-Pleistocene. Nat. Commun..

[53] Cheng H (2013). Climate change patterns in Amazonia and biodiversity. Nat. Commun..

[54] Baker PA, Fritz SC (2015). Nature and causes of Quaternary climate variation of tropical South America. Quat. Sci. Rev..

[55] Burbank DW (1996). Bedrock incision, rock uplift and threshold hillslopes in the northwestern Himalayas. Nature.

[56] Larsen IJ, Montgomery DR (2012). Landslide erosion coupled to tectonics and river incision. Nat. Geosci..

[57] Bookhagen B, Strecker MR (2012). Spatiotemporal trends in erosion rates across a pronounced rainfall gradient: Examples from the southern Central Andes. Earth Planet. Sci. Lett..

[58] DiBiase RA, Whipple KX (2011). The influence of erosion thresholds and runoff variability on the relationships among topography, climate, and erosion rate. J. Geophys. Res..

[59] Trauth MH, Bookhagen B, MARWAN N, Strecker MR (2003). Multiple landslide clusters record Quaternary climate changes in the northwestern Argentine Andes. Palaeogeogr., Palaeoclimatol., Palaeoecol..

[60] Mutz SG (2018). Estimates of late Cenozoic climate change relevant to Earth surface processes in tectonically active orogens. Earth Surf. Dyn..

[61] Marshall JA (2015). Frost for the trees: Did climate increase erosion in unglaciated landscapes during the late Pleistocene?. Sci. Adv..

[62] D’Arcy M (2019). Timing of past glaciation at the Sierra de Aconquija, northwestern Argentina, and throughout the Central Andes. Quat. Sci. Rev..

[63] Fedorov, A. V. et al. Patterns and mechanisms of early Pliocene warmth. *Nature* **496**, 43–49 (2013). 10.1038/nature12003 23552943

[64] Straub KM, Duller RA, Foreman BZ, Hajek EA (2020). Buffered, incomplete, and shredded: the challenges of reading an imperfect stratigraphic record. J. Geophys. Res.: Earth Surf..

[65] Romans BW, Castelltort S, Covault JA, Fildani A, Walsh JP (2016). Environmental signal propagation in sedimentary systems across timescales. Earth Sci. Rev..

[66] Tofelde S, Savi S, Wickert AD, Bufe A, Schildgen TF (2019). Alluvial channel response to environmental perturbations: fill-terrace formation and sediment-signal disruption. Earth Surf. Dyn..

[67] Lisiecki LE, Raymo ME, Pliocene-Pleistocene A (2005). stack of 57 globally distributed benthic δ18O records. Paleoceanography.

[68] Lease RO, Ehlers TA (2013). Incision into the Eastern Andean Plateau During Pliocene Cooling. Science.

[69] Fedorov AV (2006). The Pliocene paradox (mechanisms for a permanent El Nino). Science.

[70] Schneider T, Bischoff T, Haug GH (2014). Migrations and dynamics of the intertropical convergence zone. Nature.

[71] Kohl CP, Nishiizumi K (1992). Chemical isolation of quartz for measurement of insitu-produced cosmogenic nuclides. Geochimica et. Cosmochimica Acta.

[72] Nishiizumi K (2007). Absolute calibration of 10Be AMS standards. Nucl. Instrum. Methods Phys. Res. Sect. B: Beam Interact. Mater. At..

[73] Heisinger B (2002). Production of selected cosmogenic radionuclides by muons 1. Fast muons. Earth Planet. Sci. Lett..

[74] Heisinger B (2002). Production of selected cosmogenic radionuclides by muons: 2. Capture of negative muons. Earth Planet. Sci. Lett..

[75] Marrero SM (2016). Cosmogenic nuclide systematics and the CRONUScalc program. Quat. Geochronol..

[76] Stone JO (2000). Air pressure and cosmogenic isotope production. J. Geophys. Res.-Solid Earth.

[77] Borchers B (2016). Geological calibration of spallation production rates in the CRONUS-Earth project. Quat. Geochronol..

[78] Braucher R, Brown ET, Bourlès DL, Colin F (2003). In situ produced 10Be measurements at great depths: implications for production rates by fast muons. Earth Planet. Sci. Lett..

[79] Stap LB (2018). Modeled influence of land ice and CO_2_ on polar amplification and paleoclimate sensitivity during the past 5 million years. Paleoceanogr. Paleoclimatology.

[80] Willeit M, Ganopolski A, Calov R, Brovkin V (2019). Mid-Pleistocene transition in glacial cycles explained by declining CO_2_ and regolith removal. Sci. Adv..

[81] Brovkin V, Ganopolski A, Svirezhev Y (1997). A continuous climate-vegetation classification for use in climate-biosphere studies. Ecol. Model..

[82] Stocker TF, Mysak LA, Wright DG (1992). A zonally averaged, coupled ocean-atmosphere model for paleoclimate studies. J. Clim..

[83] Laskar J (2004). A long-term numerical solution for the insolation quantities of the Earth. Astron. Astrophys..

[84] Van de Wal RSW, de Boer B, Lourens LJ, Köhler P, Bintanja R (2011). Reconstruction of a continuous high-resolution CO2 record over the past 20 million years. Climate.

[85] Grinsted A, Moore JC, Jevrejeva S (2004). Application of the cross wavelet transform and wavelet coherence to geophysical time series. Nonlinear Process. Geophysics.

[86] Pingel H (2014). Pliocene orographic barrier uplift in the southern Central Andes. Geology.

