# Supplementary material for: Milankovitch-paced erosion in the southern Central Andes
Source: Nat Commun. 2023 Jan 26;14:424. doi: 10.1038/s41467-023-36022-0 (PMC9880006; doi:10.1038/s41467-023-36022-0)
Supplement: Supplementary file 1 — Supplementary Information [file 41467_2023_36022_MOESM1_ESM.pdf]

Milankovitch-paced erosion in the southern Central Andes

[1,2]*G. Burch Fisher, [3]Lisa V. Luna, [4]William H. Amidon, [2]Douglas W. Burbank, [5]Bas de Boer, [6]Lennert B. Stap, [7]Bodo Bookhagen, [8,9]Vincent Godard, [10]Michael E. Oskin, [11]Ricardo N. Alonso, [12]Erik Tuenter, [13]Lucas J. Lourens

AFFILIATIONS

[1] Jackson School of Geosciences, University of Texas at Austin, Austin, TX, 78712, USA
[2] Earth Research Institute, University of California, Santa Barbara, CA, 93106, USA
[3] Institute of Environmental Science and Geography, University of Potsdam, Potsdam, Germany
[4] Department of Geology, Middlebury College, Middlebury, VT, 05753, USA
[5] Earth and Climate Cluster, Faculty of Science, Vrije Universiteit Amsterdam, Amsterdam, the Netherlands
[6] Institute for Marine and Atmospheric Research, Utrecht University, Utrecht, the Netherlands
[7] Institute of Geoscience, University of Potsdam, Potsdam, Germany
[8] Aix-Marseille Univ., CNRS, IRD, INRAE, CEREGE, Aix-en-Provence, France
[9] Institut Universitaire de France, Paris, France
[10] Department of Earth and Planetary Sciences, University of California, Davis, California, USA
[11] Departamento de Geología, Universidad Nacional de Salta, Salta, Argentina
[12] Royal Netherlands Meteorological Institute (KNMI), De Bilt, the Netherlands
[13] Department of Earth Sciences, Faculty of Geosciences, Utrecht University, Utrecht, the Netherlands

*Correspondence to: gbf@ucsb.edu

SUPPLEMENTARY INFORMATION

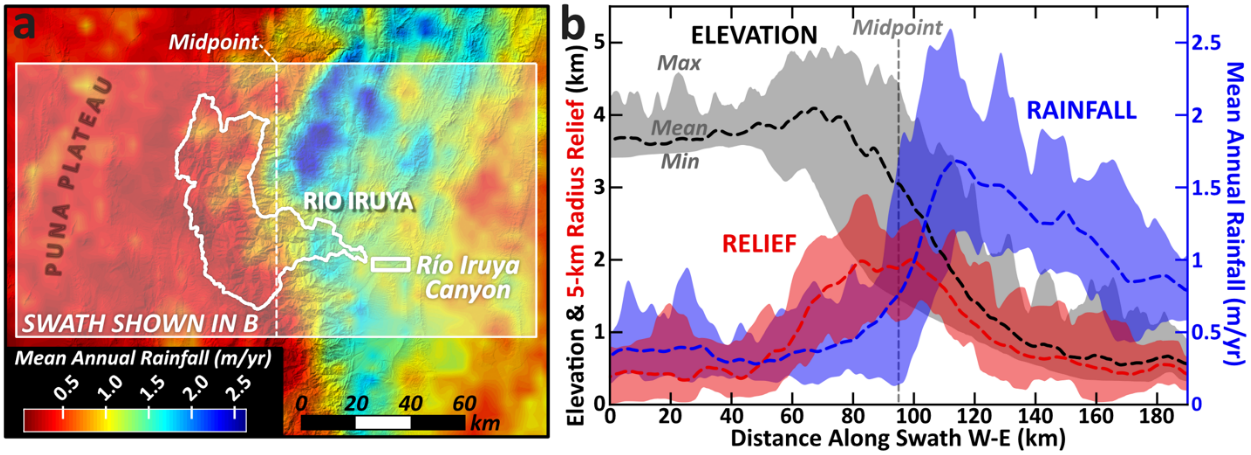

Supplementary Figure 1. Climatic and topographic characteristics of the Río Iruya watershed. (a) Mean annual rainfall in the Río Iruya region derived from Tropical Rainfall Monitoring Mission 2B31 dataset (*1*). **(b)** Results from the 100-km wide x 190-km long swath profile defined in (a) showing minimum, mean, and maximum rainfall, topography, and 5-km radius relief across the region. Topography metrics were derived from the 90-meter resolution Shuttle Radar Topography Mission (SRTM) digital elevation model.

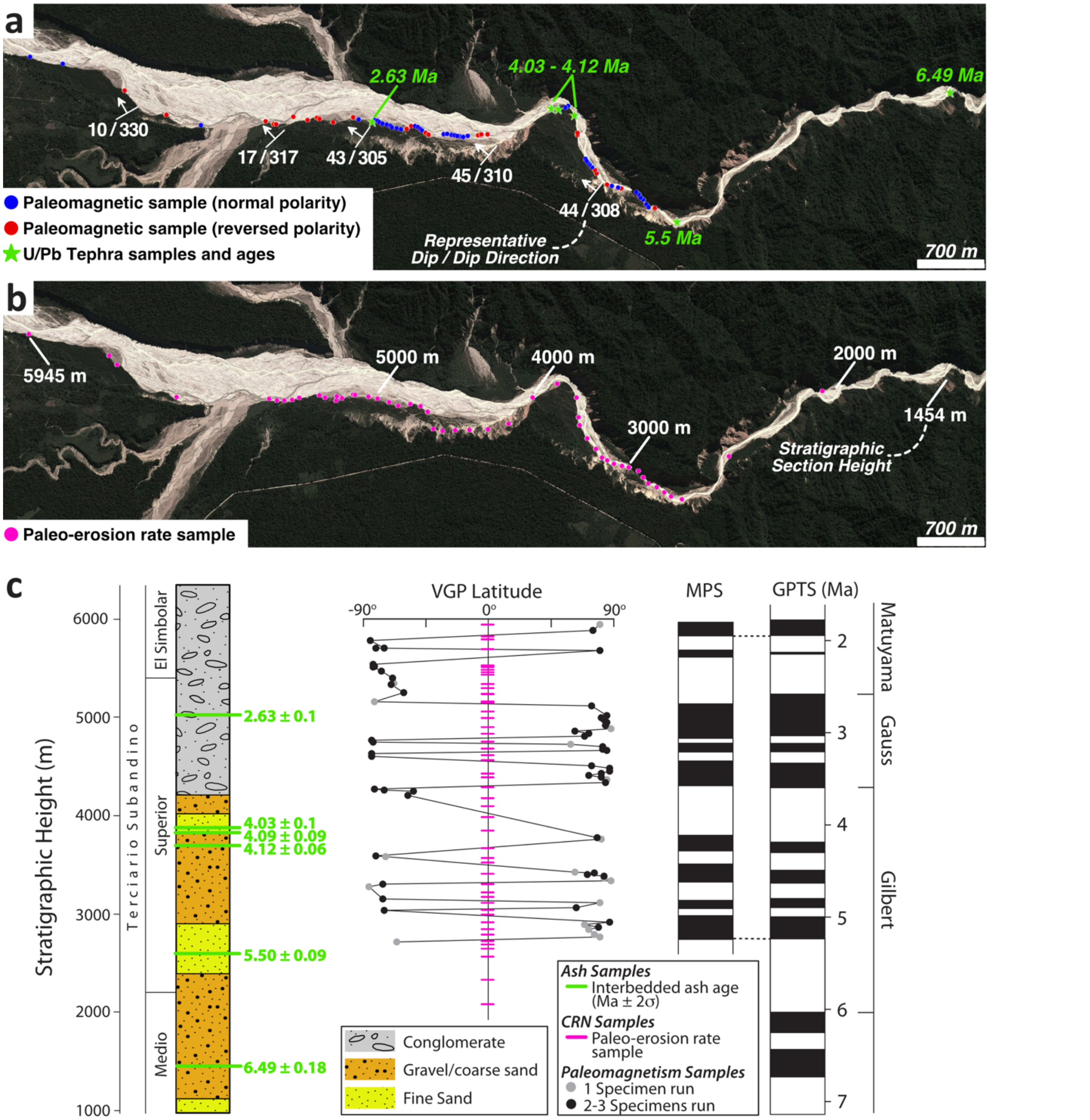

Supplementary Figure 2. Detailed sample locations and chronologic constraints for the Río Iruya section. **(a)** Google Earth imagery (image from April 2013, ©2015 DigitalGlobe) of the Río Iruya canyon showing magnetostratigraphy sample locations and polarities (red dots = reversed polarity; blue dots = normal polarity), dated tephra layers (green stars), and representative bedding dip and dip directions used to constrain paleo-erosion rate sample depositional ages (Supplementary Dataset 1). **(b)** Cosmogenic radionuclide paleo-erosion rate sample locations (magenta dots) and stratigraphic section heights along the Río Iruya canyon (Supplementary Dataset 2 and Supplementary Dataset 3). **(c)** Compilation of the stratigraphy and magnetochronology of the Río Iruya section modified after (*2*, *3*) with ash ages and paleo-erosion rate samples overlain. Note that the magnetic polarity timescale (MPS) derived from the section is compared to the global geomagnetic polarity time scale (GPTS) of (*4*). For details on the magnetostratigraphy analysis see (*2*). VGP = virtual geomagnetic pole.

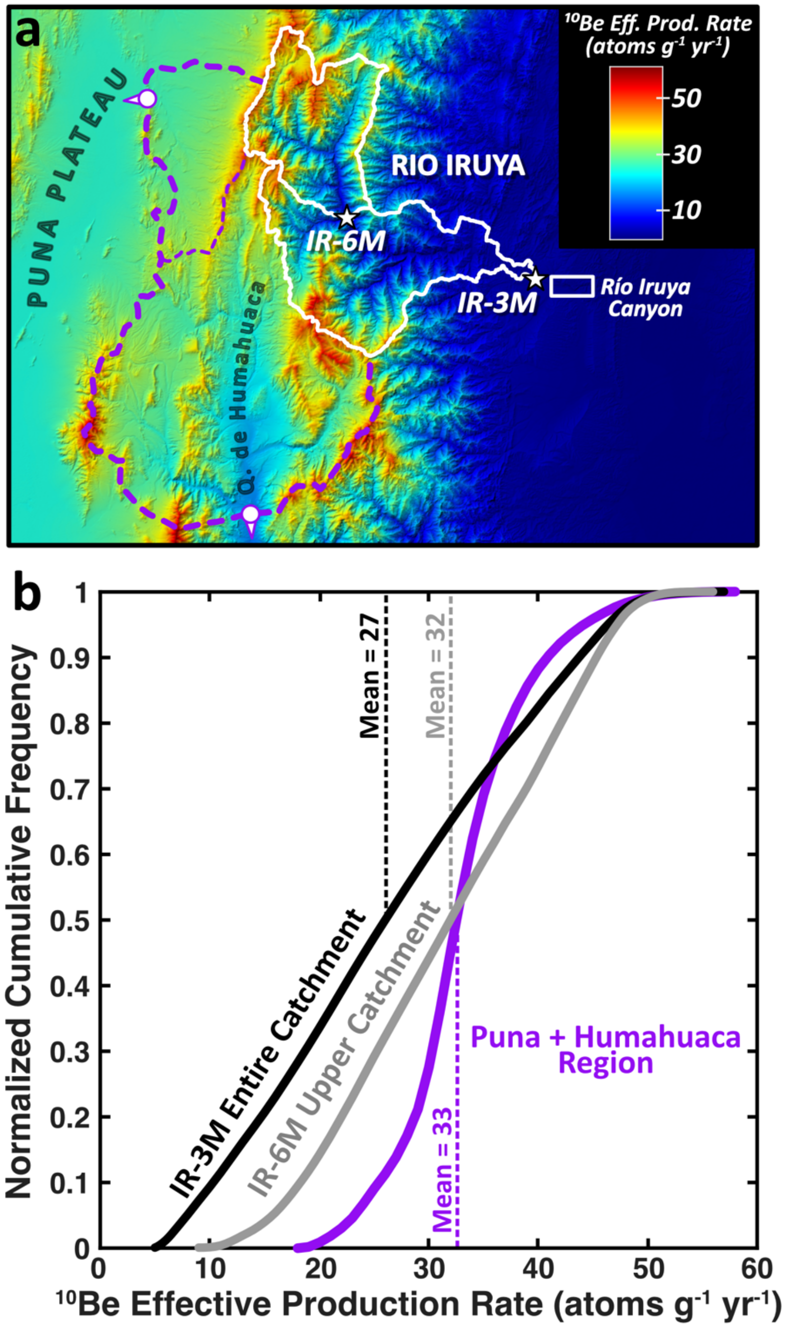

Supplementary Figure 3. Modern erosion-rate sample locations and ^{10}Be production-rate scalings. (a) Effective modern ^{10}Be production rates (scaled production rate x topographic shielding factor) for the Río Iruya study region along with modern erosion-rate sample locations (white stars) and watershed contribution areas (white outlines). Potential additional contribution area from the high-elevation upper Quebrada de Humahuaca and Puna Plateau during the period from 2.4 to ~4 Ma is delineated (hashed purple boundary) and shows present-day hydrologic pour points and flow directions (white circles and arrows). **(b)** Normalized cumulative frequency of effective production rates based on the contributing watershed hypsometry for each modern ^{10}Be erosion-rate sample, as well as the Puna Plateau and upper Quebrada de Humahuaca region shown in (a).

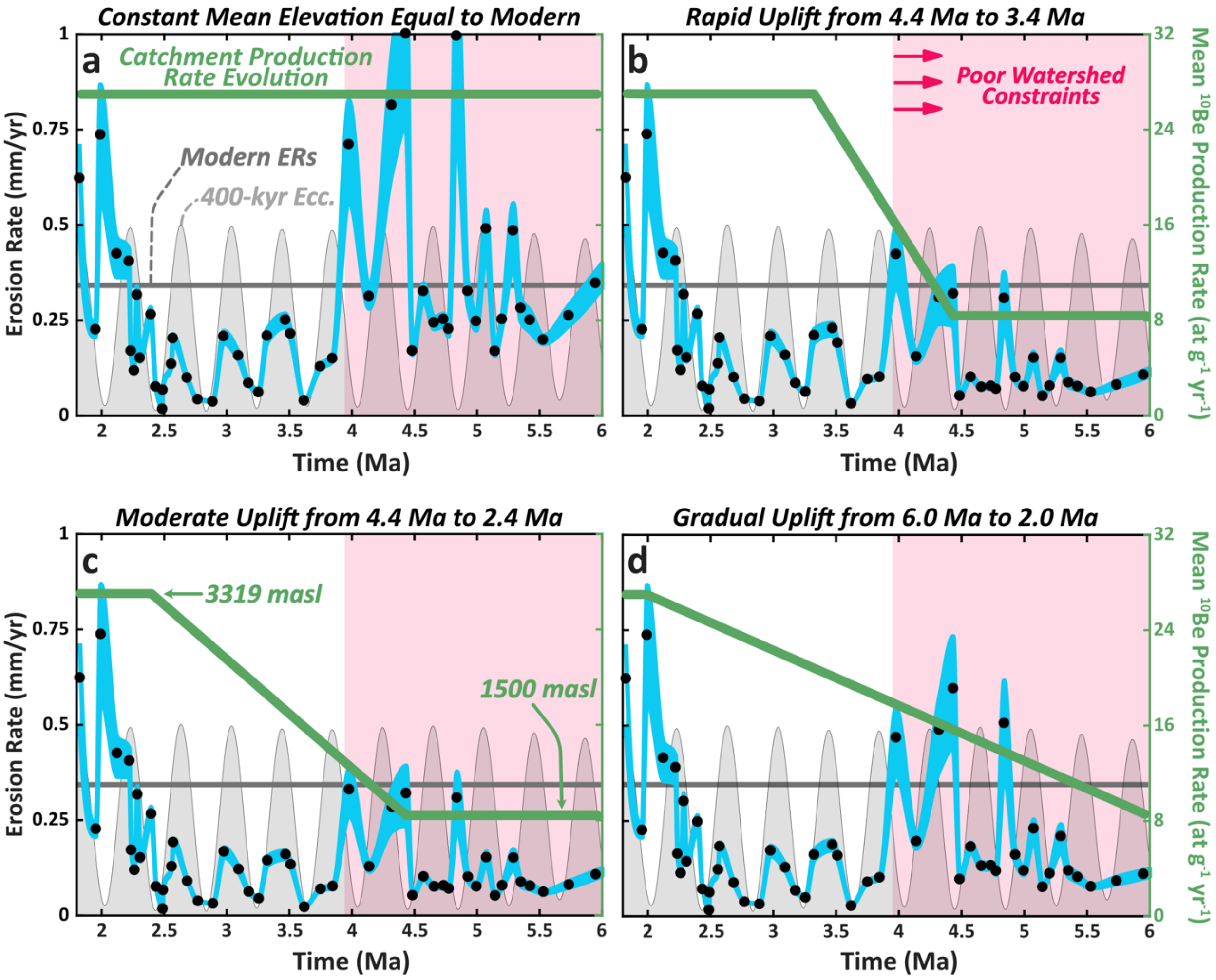

Supplementary Figure 4. Calculated ^{10}Be paleo-erosion rates from diverse mean catchment production rate scenarios. Paleo-erosion rates from the Río Iruya watershed calculated under differing catchment surface-uplift assumptions and mean production rate evolution scenarios in the contributing watershed (green). Increases in mean catchment ^{10}Be production rates are synonymous with increases in mean catchment elevation, assuming a similar hypsometry. Black circles indicate samples, and the blue piecewise cubic spline is interpolated between sample errors (±2σ envelope). Scenario **(a)** assumes a constant production rate throughout time, consistent with a constant mean elevation equal to the modern Río Iruya watershed. Scenario **(b)** and scenario **(c)** assume acceleration of Eastern Cordillera uplift around 4.5 Ma with modern mean catchment elevations reached at ~3.5 and 2.5 Ma, respectively. Scenario **(d)** assumes continuous uplift and constant mean production rate increase from 6 Ma to the modern catchment production rate at 2 Ma. All scenarios assume a final mean production rate and elevation equal to modern values of 27 at g^{-1} yr^{-1} and 3319 m elevation, respectively. Scenarios (b-d) assume initial values consistent with a lower-lying, more distal river system with mean production rate and elevation equal to 8.4 at g^{-1} yr^{-1} and 1500 m, respectively. These low initial values reflect either a similarly sized catchment to the modern Río Iruya with roughly half the relief of the modern catchment or a larger catchment with a greater proportion of drainage area at lower elevations. Scenario (b) is the preferred scenario based on regional provenance and uplift constraints and is used throughout the paper (*2, 5, 6*). Long-eccentricity cycles (400 kyr) are shown in the background for reference (light gray) with the modern Río Iruya catchment erosion rate (0.34 mm/yr) shown in dark gray.

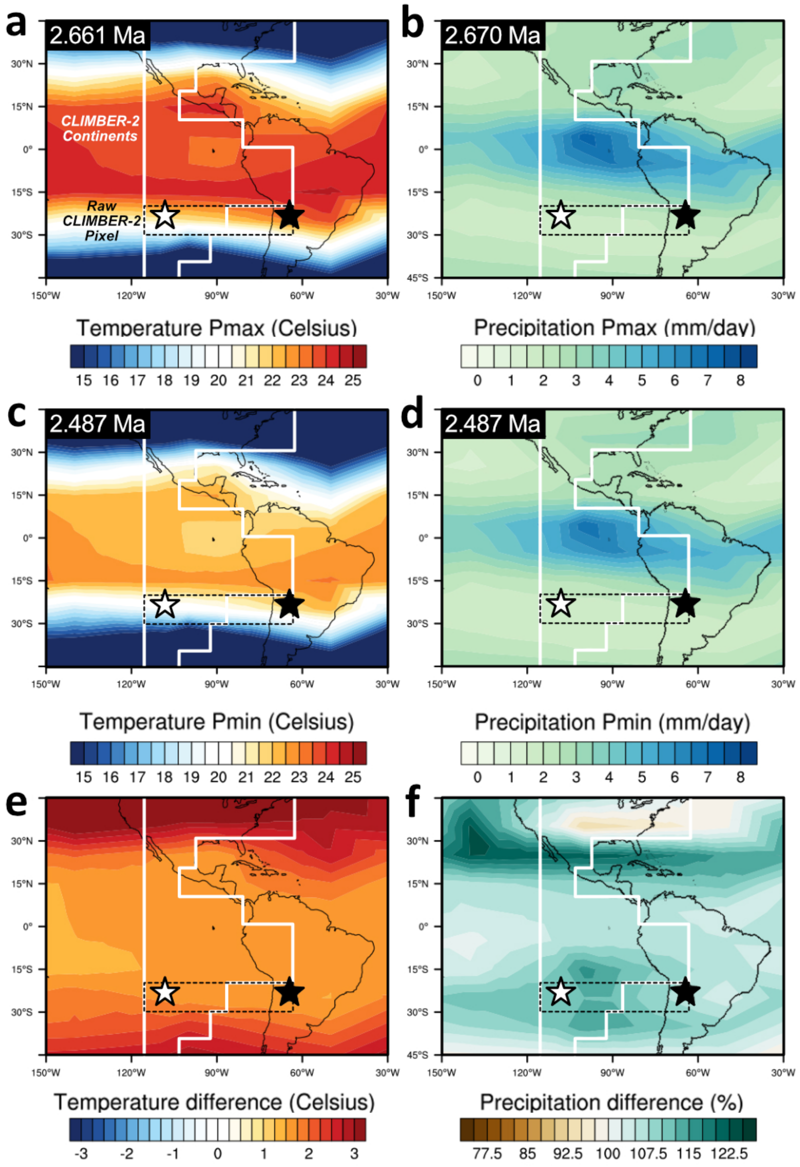

Supplementary Figure 5. CLIMBER-2 model results for precession maximum, minimum, and difference. **(a)** Temperature (°C) for a precession-driven maximum (2.661 Ma). **(b)** Precipitation (mm/day) during a precession-driven maximum (2.670 Ma). **(c)** Temperature (°C) for a precession-driven minimum (2.487 Ma). **(d)** Precipitation (mm/day) during a precession-driven minimum (2.487 Ma). **(e)** Temperature (°C) difference between (a) and (c) (Pmax – Pmin). **(f)** Precipitation percent difference between (b) and (d) (100*(Pmax/Pmin)). For plotting purposes, we have interpolated the CLIMBER-2 data from a 51.5° longitudinal resolution to a 10° longitudinal resolution using a simple linear interpolation. Note that the intent was to assess the nearest precession-driven precipitation peak/trough associated with 400-kyr eccentricity maxima and minima (Fig. 4e), which, along with the complexity of the model-derived precipitation signal, does not always align perfectly with those of the predicted insolation record. The continental setup of the model (white outline), the raw CLIMBER-2 pixel used for the study area (black dotted rectangle; 65° W - 116.5° W and 20° S - 30° S), and the approximate Río Iruya study area location within the model setup (denoted by the white star) are shown in each panel for reference. The true continental outlines (black) and study area location (black star) are shown to illustrate the spatial discrepancy between the model and reality (*7*).

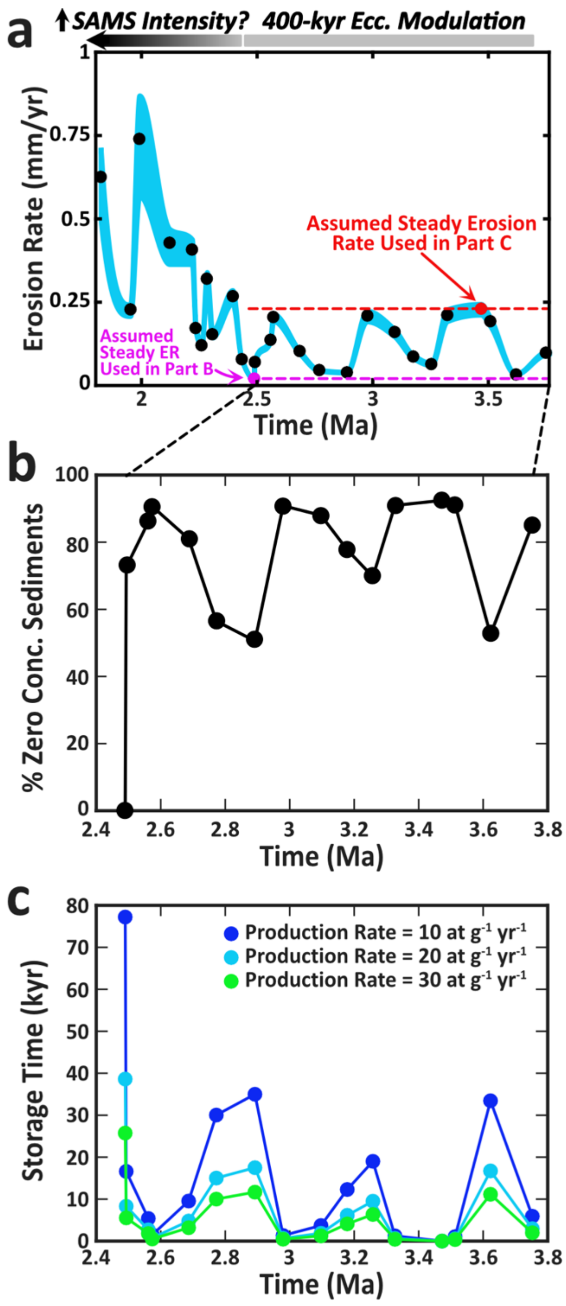

Supplementary Figure 6. End-member constraints on sediment storage times and mixing ratios under a constant erosion-rate assumption. (a) Calculated ^{10}Be derived paleo-erosion rates (±2σ envelope) from 1.8-3.8 Ma based on the preferred watershed uplift scenario (Supplementary Fig. 4b). **(b)** Influx of zero-concentration sediments necessary to produce the observed erosion-rate cyclicity shown in (a) assuming a steady basin-wide erosion rate of 0.02 mm/yr (consistent with the point at 2.49 Ma). Processes that might lead to such an influx of zero concentration sediments include deep-seated landsliding, tapping of long-buried sediments on the Puna Plateau, and glacial sediment inputs. **(c)** Sediment-storage times necessary to produce the observed erosion-rate cyclicity in (a) assuming surface exposure during storage (i.e., no burial) and a steady basin-wide erosion rate of 0.23 mm/yr (consistent with the point at 3.473 Ma). Note that analyses in (b) and (c) are only performed on actual measurements and not the interpolated spline between points shown in light blue in (a). SAMS = South American Monsoon System.

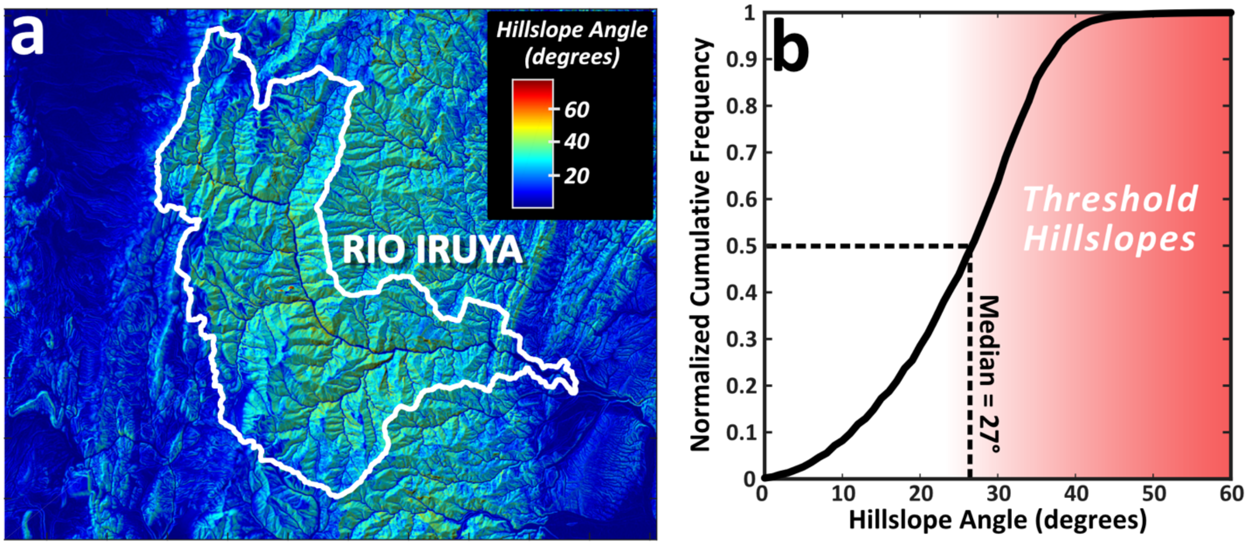

Supplementary Figure 7. Hillslope map and distribution for the Río Iruya watershed. (a) Map showing modern hillslope angles across the Río Iruya watershed along with the steep channel-margin hillslopes confining the channel network and limiting the potential for lateral sediment storage during transport. **(b)** Normalized cumulative frequency plot of hillslope angles in the Río Iruya watershed (black line) showing that ~50% of the modern catchment exceeds estimates of mean threshold hillslope values for the region (~25-30°) (red gradient) (*8*). Hillslope angles and distributions were calculated using the 90-meter resolution Shuttle Radar Topography Mission digital elevation model.

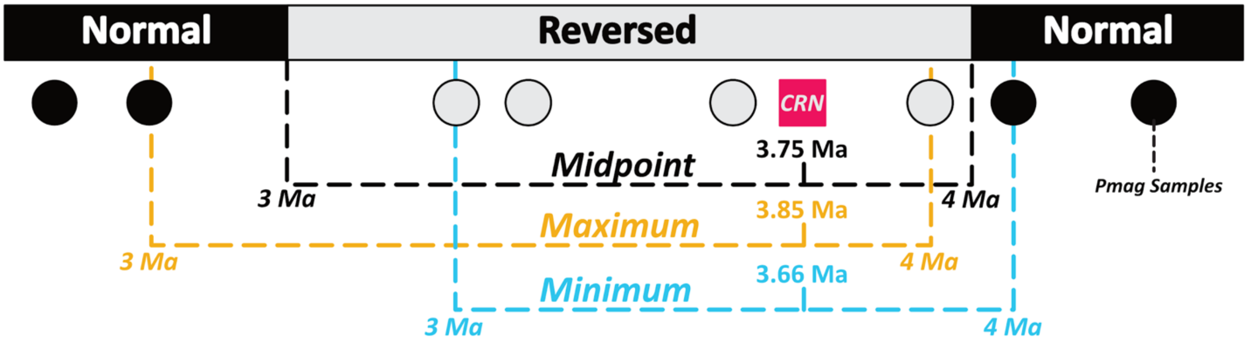

Supplementary Figure 8. Schematic of age-model error estimation. Simple schematic depicting the methodology for quantifying maximum and minimum stratigraphic age errors on each cosmogenic radionuclide sample (denoted CRN) using the magnetostratigraphic column. Note that this method allows for asymmetric error about the midpoint value and assumes a linear relationship between time and stratigraphic height between magnetic polarity reversals. Boundaries at 3 and 4 Ma are simplified for illustrative purposes and do not correspond to actual polarity reversals in the global geomagnetic polarity timescale (GPTS). Pmag = paleomagnetic.

Supplementary Table 1. Explanation of symbols and values used in the paleo-erosion rate analysis (*9*).

Symbol	Explanation	Origin of Values	^{10}Be Values	^{26}Al Values
N_A	sample concentration	measured	Supp. Dataset 2	Supp. Dataset 3
N_X	sample exhumation component	calculated	Supp. Dataset 2	Supp. Dataset 3
N_D	radioactive decay component	calculated	Supp. Dataset 2	Supp. Dataset 3
N_B	burial component	calculated	Supp. Dataset 2	Supp. Dataset 3
N_T	sediment transport component	assumed	0	0
N_E	paleo-erosion-rate component	calculated	Supp. Dataset 2	Supp. Dataset 3
εA	sample-concentration error	measured	Supp. Dataset 2	Supp. Dataset 3
εX	exhumation-component error	calculated	*Varied*	*Varied*
εD	radioactive decay error	calculated	*Varied*	*Varied*
εB	burial-component error	calculated	*Varied*	*Varied*
$\langle\varepsilon\rangle$	compounded error of paleo-erosion-rate component	calculated	Supp. Dataset 2	Supp. Dataset 3
t	age of sample	stratigraphic position	Supp. Dataset 2	Supp. Dataset 3
R_B	sample burial rate	sedimentation rate	0.06 - 0.13 cm/yr	0.06 - 0.13 cm/yr
R_E	paleo-erosion rate	calculated	Supp. Dataset 2	*N/A*
R_X	sample-exhumation rate	calculated from 1865 surface	35 - 83 cm/yr	35 - 83 cm/yr
$\boldsymbol{\delta}_t$	age error	stratigraphic position	Supp. Dataset 2	Supp. Dataset 3
$\boldsymbol{\delta}_B$	sample-burial-rate error	[a] assumed	50% or 25%	50% or 25%
ε_E	paleo-erosion-rate error	calculated	Supp. Dataset 2	Supp. Dataset 3
$\boldsymbol{\delta}_X$	exhumation-rate error	assumed	10 cm/yr	10 cm/yr
P_0	production at sample site	CRONUS calculator	4.01 at g^{-1} yr^{-1}	27.9 at g^{-1} yr^{-1}
P_B	production during burial	assumed	4.01 at g^{-1} yr^{-1}	27.9 at g^{-1} yr^{-1}
P_E	production during erosion	assumed varied scenarios	Supp. Dataset 2	*N/A*
ρs	density of sedimentary rocks	assumed	2.0 g/cm^3	2.0 g/cm^3
ρr	density of source area rocks	assumed	2.7 g/cm^3	2.7 g/cm^3
λ	decay constant	assumed	4.997 x 10^{-7} yr^{-1}	9.83 x 10^{-7} yr^{-1}
Λ	mean free path for *neutron spallation* and *slow* and *fast* muon reactions	assumed	160, 1500, 5300 g/cm^2	160, 1500, 5300 g/cm^2

[a]Assume 50% error on neutron production due to increased potential of stochastic deposition events to affect the cosmogenic signal whereas the muogenic error is integrated over a greater depth producing less variability and error (25%).

SUPPLEMENTARY REFERENCES

1. B. Bookhagen, M. R. Strecker, Orographic barriers, high-resolution TRMM rainfall, and relief variations along the eastern Andes. *Geophysical Research Letters* **35**, (2008).

2. W. H. Amidon, L. V. Luna, G. B. Fisher, D. W. Burbank, A. R. C. Kylander-Clark, R. Alonso, Provenance and tectonic implications of Orán Group foreland basin sediments, Río Iruya canyon, NW Argentina (23° S). *Basin Research* **29**, 96–112 (2015).

3. R. M. Hernández, J. Reynolds, A. Di Salvo, Análisis tectosedimentario y ubicación geocronológica del Grupo Orán en el río Iruya. *Boletín de Informaciones Petroleras* **45**, 80–93 (1996).

4. 87 L. Lourens, F. Hilgen, N. J. Shackleton, J. Laskar, D. Wilson, The neogene period. in *A Geologic Time Scale*, F. M. Gradstein, J. G. Ogg, A. G. Smith, Eds. (Cambridge University Press, Cambridge, 2004).

5. 21 H. Pingel, T. Schildgen, M. R. Strecker, H. Wittmann, Pliocene–Pleistocene orographic control on denudation in northwest Argentina. *Geology* **47**, 359–362 (2019).

6. 44 H. Pingel, M. R. Strecker, R. N. Alonso, A. K. Schmitt, Neotectonic basin and landscape evolution in the Eastern Cordillera of NW Argentina, Humahuaca Basin (~24°S). *Basin Research* **25**, 554–573 (2013).

7. V. Petoukhov, A. Ganopolski, V. Brovkin, M. Claussen, A. Eliseev, C. Kubatzki, S. Rahmstorf, CLIMBER-2: a climate system model of intermediate complexity. Part I: model description and performance for present climate. *Climate Dynamics* **16**, 1–17 (2000).

8. B. Bookhagen, M. R. Strecker, Spatiotemporal trends in erosion rates across a pronounced rainfall gradient: Examples from the southern Central Andes. *Earth and Planetary Science Letters* **327-328**, 97–110 (2012).

9. M. E. Oskin, N. E. Longinotti, T. C. Peryam, R. J. Dorsey, C. J. DeBoer, B. A. Housen, K. D. Blisniuk, Steady 10 Be-derived paleoerosion rates across the Plio-Pleistocene climate transition, Fish Creek-Vallecito basin, California. *Journal of Geophysical Research: Earth Surface* **122**, 1653–1677 (2017).
