# Supplementary material for: Milankovitch-paced erosion in the southern Central Andes
Source: Nat Commun. 2023 Jan 26;14:424. doi: 10.1038/s41467-023-36022-0 (PMC9880006; doi:10.1038/s41467-023-36022-0)
Supplement: Supplementary file 2 — Description of Additional Supplementary Files [file 41467_2023_36022_MOESM2_ESM.pdf]

Description of Additional Supplementary Files:

Supplementary Dataset 1: Paleomagnetic, ash, and sediment-accumulation rate sample information (see .xlsx file) - This file contains all paleomagnetic and ash sample information as well as sediment accumulation-rate calculations for the Río Iruya section.

Supplementary Dataset 2: ^{10}Be sample information (see .xlsx file) **-** This file contains all ^{10}Be sample information related to collection, processing, measurement, analysis, and results of modern and paleo-erosion rate calculations. Paleo-erosion rate values for scenarios (a-d) are presented and refer to the production-rate-evolution scenarios in Supplementary Fig. 4. All column headings, including units of measurement and symbol references, are defined in Supplementary Table 1. Bold ages and errors refer to those samples where age errors had to be assumed as discussed in the “Age Model and Error” section in the Methods.

Supplementary Dataset 3: ^{26}Al sample information (see .xlsx file) - This file contains all ^{26}Al sample information related to collection, processing, measurement, analysis, and concentration results of paleo-erosion rate samples. All column headings, including units of measurement and symbol references, are shown in Supplementary Table 1. Bold ages and errors refer to those samples where age errors had to be assumed as discussed in the “Age Model and Error” section in the Methods. No attempt was made to calculate paleo-erosion rates from the ^{26}Al data due to the large accelerator mass spectrometry (AMS)errors related to considerable inherent Al concentrations in the processed quartz. Inherent Al values measured immediately before quartz digestion are presented in the “Inherent Al (g)” column for reference.

Supplementary Dataset 4: CLIMBER-2 model results (see .xlsx file) **-** This file contains CLIMBER-2 model results for both time series data extracted for the study area grid cell (65° W - 116.5° W and 20° S - 30° S) and the 10° latitude x 10° longitude interpolated model results for a precession-driven maxima and minima at 2.670 Ma (2.661 Ma for temperature) and 2.487 Ma, respectively (see Supplementary Figure 5).
